# Three New Physalins from *Physalis Alkekengi* L. var. *franchetii* (Mast.) Makino

**DOI:** 10.3390/molecules30143017

**Published:** 2025-07-18

**Authors:** Ji Zhao, Xiang-Rong Zhang, You Wu, Ying-Li Liu, Yan-Feng Liang, Yang Teng

**Affiliations:** 1 College of Pharmacy, Jiamusi University, Jiamusi 154007, China; 2 College of Basic Medicine, Jiamusi University, Jiamusi 154007, China

**Keywords:** *Physalis alkekengi* L. var. *franchetii* (mast.) makino, physalins, structural identification, antitumor activity

## Abstract

*Physalis Alkekengi* L. var. *franchetii* (Mast.) Makino (PAF), which is used in both food and medicine, has a long history of about 1800 years of application in China. There are many active constituents in the calyx of PAF. Physalins and physalins with a single oxygen bridge are the unique components of the PAF calyx. Physalins with multiple biological activities, including anticancer activity, antimicrobial activity, anti-inflammatory activity, etc., have been found. As such, physalins deserve to be studied further. In this study, we aimed to extract, separate, and identify the effective components of physalins from the calyx of PAF and investigate ability to inhibit the proliferation of tumor cell lines. Three new physalins, physalin VIII (**1**), 3α-hydroxy-2,3,25,27-tetrahydro-4,7-didehydro-7-deoxyneophysalin A (**2**), and physalin IX (**3**), along with three known compounds, physalin L (**4**), physalin D (**5**), and alkekengilin A (**6**) were isolated from PAF calyxes. Physalin D was superior to the positive control drug cisplatin in inhibiting the proliferation of five tumor cell lines. The physalin compounds exhibited potential antitumor activity, being deemed worthy of further research in the fields of antitumor drug development and the application in health foods.

## 1. Introduction

Cancer is a major global health challenge. In 2022, approximately 20 million new cancer cases were diagnosed globally, with the number of cancer-related deaths reaching about 9.7 million [1]. Cancer not only decreases the likelihood of patients being able to work but also increases the economic burden on their families. The rising number of cancer patients consumes a large amount of medical resources, thus exacerbating the social burden.

There are various types of antitumor drugs used in clinical practice, but their side effects are also significant. For example, alkylating agents such as cisplatin have nephrotoxicity and are prone to developing resistance [2,3]. The use of small-molecule kinase inhibitors (e.g., gefitinib, osimertinib) is frequently associated with skin toxicity, as well as acquired resistance [4,5]. Monoclonal antibodies like trastuzumab have high specificity, but they are expensive, and most people cannot afford such treatment. Plant-derived alkaloids, such as paclitaxel, exhibit broad-spectrum anticancer activity but induce bone marrow suppression, complete alopecia, and hypersensitivity reactions [6,7].

China has abundant plant resources, meaning it could be a good source of natural antitumor drugs or lead compounds with a low adverse effect derived from plants. Therefore, for this study, a drug grown in Northeast China was chosen as the study object due to its high latitude and ability to grow in black soil, which allows for a long growth cycle and sufficient deposition of nutrients and medicinal ingredients.

*Physalis Alkekengi* L. var. *franchetii* (Mast.) Makino (PAF), also called Jin-deng-long and Hong-gu-niang, belongs to the perennial herbaceous plants of the genus *Physalis* in the Solanaceae family, renowned for its bright orange-red calyx. PAF occurs naturally across multiple regions of China, including the northeast, north, east, central, and southwest areas. Many effective components, including steroids, flavonoids, phenylpropanoids, alkaloids, and terpenoid derivatives, have been isolated from the calyx of PAF [8]. Physalins with a single oxygen bridge are the unique components of PAF calyx.

Physalins are the main active substances in PAF extracts. Physalins inhibit tumor cell proliferation. In previous studies, physalins showed a strong antitumoral effect on both non-small-cell lung cancer and multiple myeloma cells [9,10]. Physalin A and Physalin B have also been shown to inhibit the proliferation of tumor cells and induce apoptosis [11,12,13]. Physalin B also showed the most significant cytotoxic activities in [14]. In another study, withaphysalins displayed antitumor effects by suppressing the PI3K-Akt-mTOR signaling pathway [15]. Therefore, it is necessary to discover new compounds with antitumor activity in physalins.

To further explore the medicinal value of physalins, we studied ethyl acetate (EA) and petroleum ether (PE) extracts from PAF calyx, obtaining six physalins, including three novel ones. In this paper, the isolation and structural elucidation of these six compounds are described, along with their antiproliferative activity against tumor cells.

## 2. Results and Discussion

**Figure 1:**
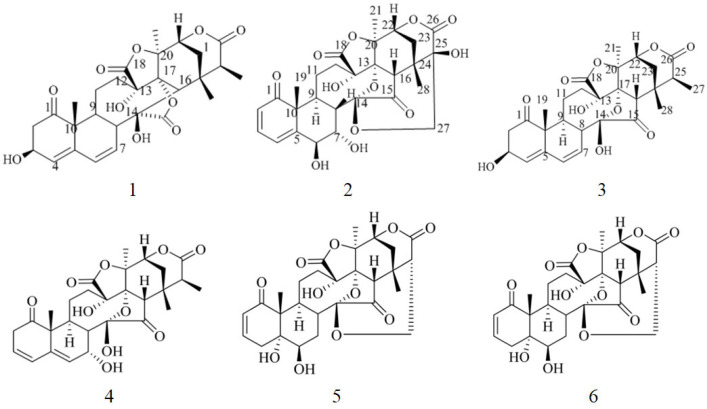
Structure of Compounds **1** to **6**.

**Figure 2:**
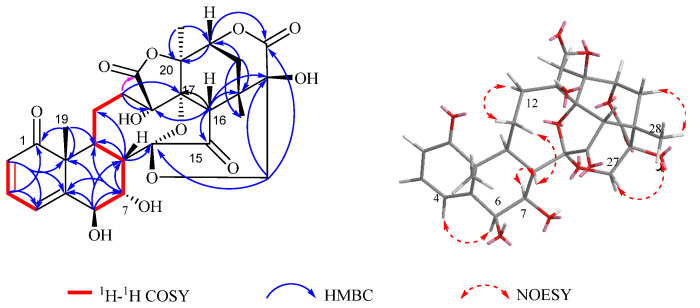
Correlation diagram of 1H-1H COSY, HMBC and NOESY of compound **1**.

Compound **1** was obtained as a white powder. Its molecular formula was established as C_28_H_30_O_12_ based on the HR-ESI-MS (*m*/*z* 581.1613 [M + Na]^+^, calcd. for C_28_H_30_O_12_Na, 581.1629), indicating 14 degrees of unsaturation. The IR spectrum exhibited absorption bands at 3356 cm^−1^, 1654 cm^−1^, and 1033 cm^−1^, suggesting the presence of hydroxyl and carbonyl groups. The ^1^H-NMR spectrum (Table 1, Supplementary Materials) observed three methyl singles at *δ*_H_ 1.37 (Me-19), 1.88 (Me-21), 1.24 (Me-28); three olefinic protons at *δ*_H_ 5.95 (H-2), 6.19 (H-4), and 7.01 (H-3); as well as three oxygen proton hydrogen signals *δ*_H_ 4.21 (H-6), 4.43 (H-7) and 4.58 (H-22). The ^13^C NMR data (Table 1) displayed a total of 28 carbons, comprising two carbonyl carbons at *δ*_C_ 209.6 (C-1) and 209.5 (C-15), four hydroxy carbonyl carbons at *δ*_C_ 77.3 (C-6), 70.4 (C-7), 80.5 (C-13) and 75.4 (C-25), one ketal carbon *δ*_C_ 108.3 (C-14), four olefinic carbons *δ*_C_ 156.2 (C-5), 141.7 (C-3), 126.7 (C-2), and 122.8 (C-4), and three methyl carbons [*δ*_C_ 20.1 (C-19), 20.1 (C-28) and 22.7 (C-21)]. The NMR spectral data of **1** closely resembled those of physalin G isolated from jin-deng-long [16], with the exception that signals of ring B and C-25 [*δ*_H_ 4.43 (1H, dd, *J* = 3.6, 1.7 Hz, H-7)]; *δ*_C_ 70.4 (C-7) and 75.4 (C-25) were present in compound **1**. These data indicated the presence of the OH group connected to C-7 in the B ring and suggested that C-25 that the C-25 was a quaternary carbon in the NMR data, which was confirmed by the key HMBC correlations from H-7 to C-6/C-8; H-6 to C-5/C-7/C-8/C-10; H-9 to C-10/C-12/C-19, as well as the key 1H-1H COSY correlations from H-7 to H-6/H-8 and H-9 to H-8/H-11 (Figure 1). The relative configuration of compound **1** was further confirmed by the nuclear Overhauser effect spectroscopy (NOESY) spectrum (Figure 2), which revealed an α-orientation of OH-6, a *β*-orientation of OH-7, and finally compound **1** was identified to be physalin VIII.

**Figure 3:**
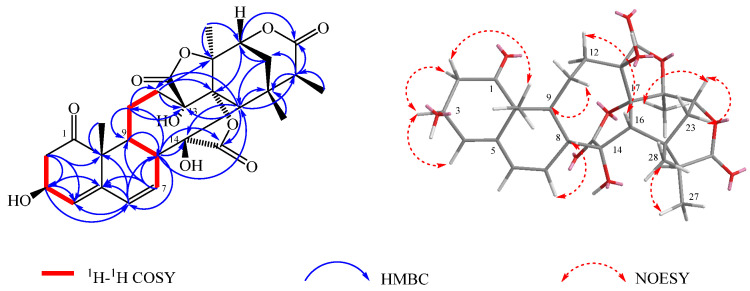
Correlation diagram of 1H-1H COSY, HMBC and NOESY of compound **2**.

Compound **2** was obtained as a white amorphous powder. Its molecular formula was established as C_28_H_32_O_10_ based on the HR-ESI-MS (*m*/*z* 551.1891 [M + Na]^+^, calcd. for C_28_H_32_O_10_Na, 551.1893), indicating 13 degrees of unsaturation. The IR spectrum exhibited absorption bands at 3357 cm^−1^, 1778 cm^−1^, and 1651 cm^−1^, suggesting the presence of hydroxyl and carbonyl groups. The ^1^H-NMR spectrum (Table 1) showed four methyl singles at *δ*_H_ 1.18 (Me-19), 1.80 (Me-21), 1.32 (3H, d, *J* = 7.5 Hz, Me-27) and 1.37 (Me-28); three olefinic protons at *δ*_H_ 5.70 (H-4), 6.19 (H-7), and 6.45 (H-6); as well as two signals of hydrogen protons at *δ*_H_ 4.52 (H-6) and 4.47 (H-7). The ^13^C NMR data (Table 1) displayed a total of 28 carbons, comprising two carbonyl carbons at *δ*_C_ 213.0(C-1), three ester carbonyl carbons at *δ*_C_ 177.6 (C-15), 176.9 (C-26) and 171.8 (C-18); three hydroxy carbonyl carbons at *δ*_C_ 68.5 (C-3), 80.5 (C-13) and 82.4 (C-14), four olefinic carbons *δ*_C_ 141.9 (C-5), 127.5 (C-4), 131.0 (C-6) and 129.6 (C-7), and four methyl carbons *δ*_C_ 20.5 (C-19), 20.8 (C-21), 28.7 (C-28) and 17.4 (C-27). The NMR spectral data of **2** closely resembled those of physalin II isolated from jin-deng-long [17], with the exception that the signals of ring A lacked a methoxy signal and the chemical shift of δ_C_ 68.5 (C-3) was 6 ppm lower than that of physalin II. It is presumed that the substitution at C-3 of compound **2** was oxidized from a methoxy group to a hydroxy group. These data indicated the presence of a hydroxyl group connected to C-3 in the A ring according to the NMR data, which was confirmed by the key HMBC correlations from H-2 to C-3/C-4/C-10; H-3 to C-4/C-5; H-7 to C-5/C-8/C-9, as well as the key ^1^H-^1^H COSY correlations from H-3 to H-2 and H-4 to H-3 (Figure 1). The relative configuration of compound **2** was further confirmed by the NOESY spectrum (Figure 3), which revealed an α-orientation of OH-3. Accordingly, compound **2** was identified as 3*α*-hydroxy-2,3,25,27-tetrahydro-4,7-didehydro-7-deoxyneo physalin A.

**Figure 4:**
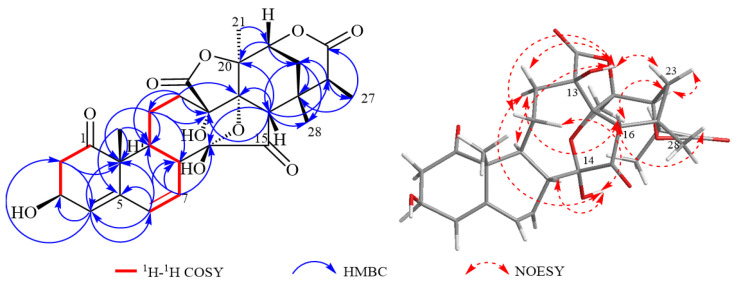
Correlation diagram of 1H-1H COSY, HMBC, and NOESY of compound **3**.

Compound **3** was obtained as a colorless needle crystal. Its molecular formula was established as C_28_H_32_O_10_ based on the HR-ESI-MS (*m*/*z* 551.1893 [M + Na]^+^, calcd. for C_28_H_32_O_10_Na, 551.1898), indicating 13 degrees of unsaturation. The IR spectrum exhibited absorption bands 3323 cm^−1^, 1769 cm^−1^ and 1700 cm^−1^, suggesting the presence of hydroxyl and carbonyl groups. The ^1^H-NMR spectrum (Table 1) observed four methyl singles at *δ*_H_ 1.39 (Me-19), 1.91 (Me-21), 1.32 (d, *J* = 7.2 Hz, Me-27) and 1.34 (Me-28); three olefinic protons at *δ*_H_ 5.61 (H-4), 6.17 (H-6), and 6.33 (H-7); as well as two oxygen proton hydrogen signals *δ*_H_ 4.57 (H-22), and 4.67 (H-3). The ^13^C NMR data (Table 1) displayed a total of 28 carbons, comprising two carbonyl carbons at *δ*_C_ 211.7 (C-1) and 212.5 (C-15), one hydroxy carbonyl carbon at *δ*_C_ 69.6 (C-3), one ketal carbon *δ*_C_ 101.7 (C-14), four olefinic carbons at *δ*_C_ 142.1 (C-5), 128.0 (C-6), 127.9 (C-7) and 126.0 (C-4), and four methyl carbons *δ*_C_ 20.5 (C-19), 20.8 (C-21), 16.3 (C-27) and 26.1 (C-28). The NMR spectral data of **3** closely resembled those of physalin L isolated from the plant [18], with the exception that signals of ring A [*δ*_H_ 4.67 (1H, s, H-3)]; *δ*_C_ 69.6 (C-3) was present in compound **3**. These data indicated the presence of the OH connected to C-3 in the A ring in the NMR data, which was confirmed by the key HMBC correlations from H-4 to C-2/C-3/C-10; H-2 to C-3/C-4/C-10; H-6 to C-4/C-5/C-10; H-7 to C-5/C-6/C-8/C-14; H-9 to C-10/C-12/C-19, as well as the key ^1^H-^1^H COSY correlations from H-6 to H-7; H-7 to H-8 and H-9 to H-8/H-11 (Figure 1). It turns out that the olefin double bond position is C-4/C-5 and C-6/C-7. The relative configuration of compound **3** was further confirmed by the NOESY spectrum (Figure 4), which δ_H_ 2.86 (H-2) was correlated with 13-OH, 14-OH was correlated with δH 1.61 (H-11), δ_H_ 1.81 (H-12), and δ_H_ 2.72 (H-8), and 13-OH was correlated with δH 2.19 (H-23), δ_H_ 2.99 (H-9), and δ_H_ 2.43 (H-12), and it can be deduced that 3-OH and 13-OH are α-configurations and 14-OH is a β configuration. OH and 13-OH are in α-configuration and 14-OH is in β-configuration. Hence, compound **3** was identified as being physalin Ⅸ.

Compounds **4** to **6** (Figure 1) were determined to be physalin L [19], physalin D [20], and alkekengilin A [21].

Compounds **1** to **6** were examined for their cytotoxicity against HL-60, A-549, SMMC-7221, MDA-MB231, and SW480 cancer cell lines. Initial screening of the six isolated compounds showed that compound **5** inhibited the growth of five tumor cells by more than 50%, as shown in Table 2. Compound **5** was subjected to a gradient rescreening, which showed that it inhibited the growth of these five cells with IC_50_ values of 0.0520, 2.274, 1.080, 0.994, and 0.649 μM, as shown in Table 3. Compound **5** exhibits a significant difference in inhibitory activity between tumor cells and normal cells (BEAS-2B), with a stronger selective inhibitory effect on tumor cells. All five antitumor activities were superior to cisplatin when compared to the positive control drug. Overall, Paclitaxel showed a significantly stronger inhibitory activity on most tumor cells (such as HL-60, A-549, etc.) than Compound **5** (*p* < 0.01), but it also had a significantly higher toxicity to normal BEAS-2B cells (*p* < 0.01). Although the tumor inhibiting activity of Compound **5** is relatively moderate, it shows better safety in normal cells. This statistical difference between tumor-inhibition and normal cell toxicity clearly distinguishes the action characteristics of the two compounds and provides a basis for the subsequent screening of highly safety antitumor compounds.

## 3. Materials and Methods

### 3.1. General Experimental Procedures

NMR spectra were obtained by a Bruker Avance III HD 600 NMR Spectrometer (Bruker, Ettlingen, Germany). UPLC/MS/MS data were acquired on a UPLC-Q Exactive MS (Thermo, New York, NY, USA). HR-ESI-MS were measured by a Xevo TQ-Smicro MS/MS mass spectrometer (Waters, New York, NY, USA). HPLC was analyzed using Waters 1525 HPLC (Waters, New York, NY, USA) and YMC Triart C_18_ column (5 μm, 10 × 250 mm). UV spectra were measured by a JASCO J-815 CD spectrometer (JASCO Co., Ltd., Hitachi, Japan). FT-IR spectra were recorded on a Thermo NICOLET 6700 advanced Fourier transform infrared spectrometer (Thermo, New York, NY, USA). Optical rotations were measured on a MCP500 high-precision digital polarimeter (Anton Paar, Graz, Austria). ODS gel (45 μm, YMC Co., Ltd., Sakyo-ku, Japan), silica gel (Qingdao Haiyang Chemical Co., Ltd., Qingdao, China), and Sephadex LH-20 (GE Healthcare Bio-Sciences AB, Uppsala, Sweden) were used for column chromatography. All cell lines (HL-60, A-549, SMMC-7221, MDA-MB-231, SW480 and BEAS-2B) were obtained from the Cell Bank of the Chinese Academy of Sciences (Beijing, China).

### 3.2. Plant Material

The calyxes of PAF were collected from Jiamusi city, Heilongjiang province in July 2019 (coordinates 130°22′08″ E and 46°48′35″ N), and were identified by Professor Lihong Wang (School of Pharmacy, Jiamusi University). The sample was stored in the School of Pharmacy, Jiamusi University.

### 3.3. Extraction and Isolation

The desiccated calyxes of PAF (16 kg) were extracted with 95% ethanol (10:1 *v*/*v*) three times, at 60 °C for 3 h each time, and concentrated into a paste swing. The extraction was extracted with PE, Dichloromethane, EA, and n-Butanol successively. The EA extraction (88.87 g) was purified by silica gel column (SGC) into four fractions (Fr.1–Fr.4) with CH_2_Cl_2_/MeOH (50:1 to 0:1, *v*/*v*).

Fr.2 (10.5 g) was separated into two fractions (Fr.2.1–Fr.2.2) by SGC. Fr.2.1 (8.3 g) was made into Fr.2.1.1 by normal phase silica gel, reversed-phase C_18_, and Sephadex LH-20. Compound **1** (2.7 mg, t_R_ = 23.5 min) was obtained from Fr.2.1.1 (69.0 mg) by HPLC (MeOH-H_2_O, 55:45, 2 mL/min). Fr.3 (13.9 g) was separated into four fractions (Fr.3.1–Fr.3.4) by ODS reversed-phase CC using a gradient elution method (30%, 40%, 60%, 75%, 80%, 90% MeOH). Compound **2** (2.3 mg, t_R_ = 28.0 min) was obtained from Fr.3.1 (18.7 mg) by HPLC (MeOH-H_2_O, 65:35, 2 mL/min).

The PE extract (164.2 g) was separated into 2 fractions (Fr.5–Fr.6) by silica gel CC with CH_2_Cl_2_/MeOH (50:1 to 0:1). Fr. 5 (18.9 g) was purified by ODS reversed-phase CC to obtain 2 fractions Fr.5.1 and Fr.5.2. Fr.5.1 (320 mg) was purified by Sephadex LH-20 (MeOH) to obtain the fraction Fr.5.1.1. Compound **3** (1.7 mg, t_R_ = 20.5 min) was isolated from Fr.5.1.1 (7.8 mg) by HPLC (MeCN-H_2_O, 35:65, 2 mL/min). Fr.5.2 (189.7 mg) was purified by Sephadex LH-20 (MeOH) to obtain two fractions Fr.5.2.1 and Fr.5.2.2. Compound **4** (16.8 mg, t_R_ = 26.5 min) was obtained from Fr.5.2.1 (36.7 mg) by HPLC (MeOH-H_2_O, 35:65, 2 mL/min). Compound **5** (11.1 mg, t_R_ = 32.8 min) was obtained from Fr. 5.2.2 (43.7 mg) by HPLC (MeOH-H_2_O, 52:48, 2 mL/min). Fr. 6 (12.8 g) was isolated into three fractions Fr. 6.1, Fr. 6.2, and Fr. 6.3 by silica gel CC, ODS reversed-phase CC, and Sephadex LH-20. Compound **6** (3.3 mg, t_R_ = 33.0 min) was purified from Fr.6.1 (60.8 mg) by HPLC (MeOH-H_2_O, 52:48, *v*/*v*, 2 mL/min).

### 3.4. Description of Spectral Data

Physalin VIII (compound **1**): Colorless crystals (CD_3_OD); [α]20 D 2062.9 (c 1.0, MeOH). IR (KBr) ν_max_ 3356, 2945, 1654, 1033 cm^−^^1^; UV(MeOH) λ_max_ 203 nm, 311 nm; ECD(MeOH) Δɛ_202nm_ −0.4322, Δɛ_213nm_ +1.5088, Δɛ_313nm_ −4.6029, Δɛ_369nm_ +2.2255; ^1^H NMR (CD_3_OD, 600 MHz), and ^13^C NMR (CD_3_OD, 150 MHz) data see Table 1; HR-ESI-MS: *m*/*z* 581.1612 [M + Na]^+^ (calcd for C_28_H_32_O_12_Na, 581.1629).

3*α*-hydroxy-2,3,25,27-tetrahydro-4,7-didehydro-7-deoxyneophysalin A (compound **2**): Colorless crystals (CD_3_OD); [α]20 D 545.0 (c 1.0, MeOH). IR (KBr) ν_max_ 3357, 2922, 1778, 1651 cm^−^^1^; UV(MeOH) λ_max_ 227 nm, 245 nm; ECD(MeOH) Δɛ_208nm_ +0.3977, Δɛ_223nm_ −0.2956, Δɛ_240nm_ +0.3671, Δɛ_263nm_ −0.2347; ^1^H NMR (CD_3_OD, 600 MHz), and ^13^C NMR (CD_3_OD, 150 MHz) data see Table 1; HR-ESI-MS: m/z 551.1891 [M + Na]^+^ (calcd for C_28_H_32_O_10_Na, 551.1893).

Physalin Ⅸ (compound **3**): Colorless crystals (CD_3_OD); [α]20 D −568.0 (c 1.0, MeOH). IR (KBr) ν_max_ 3323, 1769, 1700, 1063 cm^−^^1^; UV(MeOH) λ_max_ 209 nm, 223 nm; ECD(MeOH) Δɛ_207nm_ +3.4543, Δɛ_247nm_ −6.2571, Δɛ_226nm_ −2.8912, Δɛ_279nm_ +0.2172; ^1^H NMR (CD_3_OD, 600 MHz), and ^13^C NMR (CD_3_OD, 150 MHz) data see Table 1; HR-ESI-MS: *m*/*z* 551.1893 [M + Na]^+^ (calcd for C_28_H_32_O_10_Na, 551.1898).

### 3.5. In Vitro Antitumor Cell Proliferation Assay

The same method was used to detect cancer cell activity [22]. Five tumor cell lines (HL-60, A-549, SMMC-7221, MDA-MB-231 and SW480) were maintained in DMEM containing 10% fetal bovine serum and 0.29 g/L L-glutamine. All cells were obtained from American Type Culture Collection and incubated at 37 °C in a moist environment containing 5% CO_2_.

The compounds were dissolved in 0.1% DMSO or deionized sterile water, and the monomeric compounds were primed at a concentration of 40 μM, with a final volume of 200 μL per well, and three replicate wells were set up for each treatment.

After 48 h of incubation at 37 degrees Celsius, the wall cells were incubated for 24 h with 20 μL of MTS solution and 100 μL of culture medium; the suspension cells HL-60 were incubated for 2~4 h with 100 μL of culture supernatant, and 20 μL of MTS solution was added into each well. Parallel solvent control wells containing 0.1% DMSO in culture medium (without test compounds) were included in all assays. Three blank wells (a mixture of 20 μL of MTS solution and 100 μL of culture medium) were established, followed by 2~4 h incubation for complete reaction. After that, the light absorption value was determined. The wavelength of 492 nm was selected, and the absorbance value of each well was read by a multifunctional enzyme labeling instrument (MULTISKAN FC, Waltham, MA, USA), and the results were recorded. The compounds with half inhibition were re-screened, and two positive compounds, Cisplatin and Paclitaxel, were set, and the IC_50_ values of the compounds were calculated by the Reed and Muench method.

## Figures and Tables

**Table 1:** NMR spectroscopic data for compounds **1**–**2** in MeOH and **3** in CDCl_3_.

NO.	1		2		3	
	*δ*_C_	*δ*_H_	*δ*_C_	*δ*_H_	*δ*_C_	*δ*_H_
1	209.6		213.0		211.7	
2	126.7	5.95 (d, *J* = 9.7 Hz, 1H)	47.75	2.77 (dd, *J* = 12.6, 5.2 Hz, 1H) 2.69 (dd, *J* = 12.6, 8.7 Hz, 1H)	44.9	2.86 (dd, *J* = 12.5, 8.7 H-z, 1H) 3.10 (dd, *J* = 12.5, 7.2 H-z, 1H)
3	141.7	7.01 (dd, *J* = 9.7, 5.9 Hz, 1H)	68.5	4.47 (t, *J* = 6.12 Hz, 1H)	69.6	4.67 (s, 1H)
4	122.8	6.19 (d, *J* = 5.9 Hz, 1H)	127.5	5.70 (d, *J* = 1.20 Hz, 1H)	126.0	5.61 (d, *J* = 3.4 H-z, 1H)
5	156.2		141.9		142.1	
6	77.3	4.21 (d, *J* = 3.6 Hz, 1H)	131.0	6.45 (d, *J* = 10.41 Hz, 1H)	128.0	6.17 (dd, *J* = 10.4, 2.8 H-z, 1H)
7	70.4	4.43 (dd, *J* = 3.6, 1.7 Hz, 1H)	129.6	6.19 (dd, *J* = 10.41, 4.38 Hz, 1H)	127.9	6.33 (d, *J* = 10.4 H-z, 1H)
8	44.3	2.67 (dd, *J* = 11.6, 1.7 Hz, 1H)	50.1	2.90 (t, *J* = 5.18 Hz, 1H)	45.2	2.74 (d, *J* = 11.2 H-z, 1H)
9	33.7	3.26 (ddd, *J* = 11.6, 9.2 Hz, 1H)	38.5	1.97 (m, 1H)	31.7	3.01 (dd, *J* = 11.2, 3.6 H-z, 1H)
10	54.9		51.5		51.5	
11	22.6	2.74 (ddd, *J* = 16.5, 13.0, 1.7 Hz, *α*-H),1.16 (dddd, J = 16.5, 10.3, 9.2, 6.4 Hz, *β*-H)	24.8	1.97 (m, 1H), 1.49 (m, 1H)	24.8	1.61 (m, 1H), 1.15 (m, 1H)
12	26.5	1.49 (ddd, *J* = 16.8, 10.3, 1.7 Hz, *α*-H) 2.12 (ddd, *J* = 16.8, 13.0, 6.4 Hz, *β*-H)	29.2	2.46 (m, 1H) 2.12 (dd, *J* = 15.1, 4.2 Hz, 1H)	29.2	1.81 (m, 1H), 2.45 (m, 1H)
13	80.5		80.5		80.7	
14	108.3		82.4		101.7	
15	209.5		177.6		212.5	
16	55.8	2.70 (s, 3H)	58.2	2.96 (s, 1H)	54.8	2.45 (m, 1H)
17	82.6		82.5		82.5	
18	173.5		171.8		171.8	
19	20.1	1.37 (s, 3H)	20.5	1.18 (s, 3H)	20.5	1.39 (s, 3H)
20	81.3		83.1		83.1	
21	22.7	1.88 (s, 3H)	20.8	1.80 (s, 3H)	20.8	1.91 (s, 3H)
22	79.4	4.58 (dd, *J* = 3.6, 2.0 Hz, 1H)	78.7	4.52 (dd, *J* = 4.2, 1.61 Hz, 1H)	78.7	4.57 (d, *J* = 4.0 Hz, 1H)
23	29.8	2.50 (dd, *J* = 14.7, 3.6 Hz, α-H) 1.76 (d, *J* = 14.7, 2.0 Hz, *β*-H)	30.6	2.13 (dd, *J* = 15.1, 4.2 Hz, 1H), 1.66 (d, *J* = 15.1 Hz, 1H)	30.6	2.19 (dd, *J* = 15.1, 4.0 Hz, 1H) 1.76 (d, *J* = 15.1 Hz, 1H)
24	37.3		35.7		35.7	
25	75.4		41.0	3.63 (m, 1H)	42.8	2.72 (d, *J* = 7.2, 1.5 Hz, 2H)
26	172.1		176.9		176.7	
27	66.6	4.13 (d, *J* = 12.8 Hz, *α*-H) 3.60 (d, *J* = 12.8 Hz, *β*-H)	17.4	1.32 (d, *J* = 7.5 Hz, 3H)	16.3	1.32 (d, *J* = 7.2, 3H)
28	20.1	1.24 (s, 3H)	28.7	1.37 (s, 3H)		

**Table 2:** Cytotoxicity test results of picrin compounds (inhibition rate %).

Compound	HL-60	A-549	SMMC-7221	MDA-MB-231	SW480
**1**	18.04 ± 1.71	2.92 ± 0.84	11.68 ± 1.96	19.62 ± 1.30	15.70 ± 1.50
**2**	14.38 ± 0.86	1.69 ± 1.12	0.42 ± 3.12	15.20 ± 1.04	11.70 ± 1.24
**3**	4.03 ± 1.93	1.84 ± 2.11	11.38 ± 1.47	7.94 ± 1.61	13.18 ± 2.04
**4**	3.55 ± 0.69	12.57 ± 0.47	30.37 ± 0.60	22.23 ± 1.47	20.31 ± 2.22
**5**	106.29 ± 0.13	98.16 ± 0.29	99.66 ± 0.11	96.14 ± 0.95	99.05 ± 0.04
**6**	12.47 ± 1.75	11.10 ± 0.07	29.71 ± 0.98	9.68 ± 2.19	12.94 ± 2.18

**Table 3:** Inhibitory effects of plasmonins on proliferation of five tumor cell lines (IC50, μM).

Compound	HL-60	A-549	SMMC-7221	MDA-MB-231	SW480
Compound **5**	0.0520 ± 0.0037 **^,##^	2.274 ± 0.090 **^,##^	1.080 ± 0.027 **^,##^	0.994 ± 0.035 **^,##^	0.649 ± 0.027 **^,##^
Cisplatin	5.865 ± 0.500 **^,##^	18.91 ± 0.44 **	7.819 ± 0.405 **^,##^	21.68 ± 1.56 **^,##^	20.35 ± 0.37 **^,##^
Paclitaxel	<0.008 **^,##^	<0.008 **	0.029 ± 0.003 **^,##^	<0.008 **^,##^	<0.008 **^,##^

Note: Intra-group comparison ** *p* < 0.01. Inter-group comparison ^##^ *p*< 0.01.

## Data Availability

Calyxes of *Physalis Alkekengi* L. var. *franchetii* (Mast.) Makino were used in this study. These were purchased from farmers in Jiamusi City and its surrounding areas.

## Supplementary Materials

The following supporting information can be downloaded at: https://www.mdpi.com/article/10.3390/molecules30143017/s1, Figure S1: ^1^H NMR spectrum (600 MHz) of compound **1** in CD_3_OD; Figure S2: 13C NMR spectrum (150 MHz) of compound **1** in CD_3_OD; Figure S3: DEPT spectrum (135°) of compound **1** in CD_3_OD; Figure S4: 1H-1H COSY spectrum of compound **1** in CD_3_OD; Figure S5: HSQC spectrum of compound **1** in CD_3_OD; Figure S6: HMBC spectrum of compound **1** in CD_3_OD; Figure S7: NOSEY spectrum of compound **1** in CD3OD; Figure S8: HR-ESI-MS spectrum of compound **1**; Figure S9: IR spectrum of compound **1**; Figure S10: 1H NMR spectrum (600 MHz) of compound **2** in CD_3_OD; Figure S11: 13C NMR spectrum (150 MHz) of compound **2** in CD_3_OD; Figure S12: DEPT 135 spectrum of compound **2** in CD_3_OD; Figure S13: 1H-1H COSY spectrum of compound **2** in CD_3_OD; Figure S14: HSQC spectrum of compound **2** in CD_3_OD; Figure S15: HMBC spectrum of compound **2** in CD_3_OD; Figure S16: NOSEY spectrum of compound **2** in CD_3_OD; Figure S17: HR-ESI-MS spectrum of compound **2**; Figure S18: IR spectrum of compound **2**; Figure S19: 1H NMR spectrum (600 MHz) of compound **3** in CDCl_3_; Figure S20: 13C NMR spectrum (150 MHz) of compound **3** in CDCl_3_; Figure S21: DEPT 135 spectrum of compound **3** in CDCl_3_; Figure S22: 1H-1H COSY spectrum of compound **3** in CDCl_3_; Figure S23: HSQC spectrum of compound **3** in CDCl_3_; Figure S24: HMBC spectrum of compound **3** in CDCl_3_; Figure S25: NOSEY spectrum of compound **3** in CDCl_3_; Figure S26: HR-ESI-MS spectrum of compound **3**; Figure S27: IR spectrum of compound **3**; Figure S28: 1H NMR spectrum (600 MHz) of compound **4** in CD_3_OD; Figure S29: 13C NMR spectrum (150 MHz) of compound **4** in CD_3_OD; Figure S30: 1H NMR spectrum (600 MHz) of compound **5** in CDCl_3_; Figure S31: 13C NMR spectrum (150 MHz) of compound **5** in CDCl_3_; Figure S32: 1H NMR spectrum (600 MHz) of compound **6** in CDCl_3_; Figure S33: ^13^C NMR spectrum (150 MHz) of compound **6** in CDCl_3_.

## Abbreviations

The following abbreviations are used in this manuscript:

PAF	*Physalis Alkekengi* L. var. *franchetii* (Mast.) Makino
PE	petroleum ether
EA	ethyl acetate
SGC	silica gel column
