# Supplementary material for: Three New Physalins from Physalis Alkekengi L. var. franchetii (Mast.) Makino
Source: Molecules. 2025 Jul 18;30(14):3017. doi: 10.3390/molecules30143017 (PMC12299125; doi:10.3390/molecules30143017)
Supplement: Supplementary file 1 [file molecules-30-03017-s001.zip › molecules-3744041-supplementary.pdf]

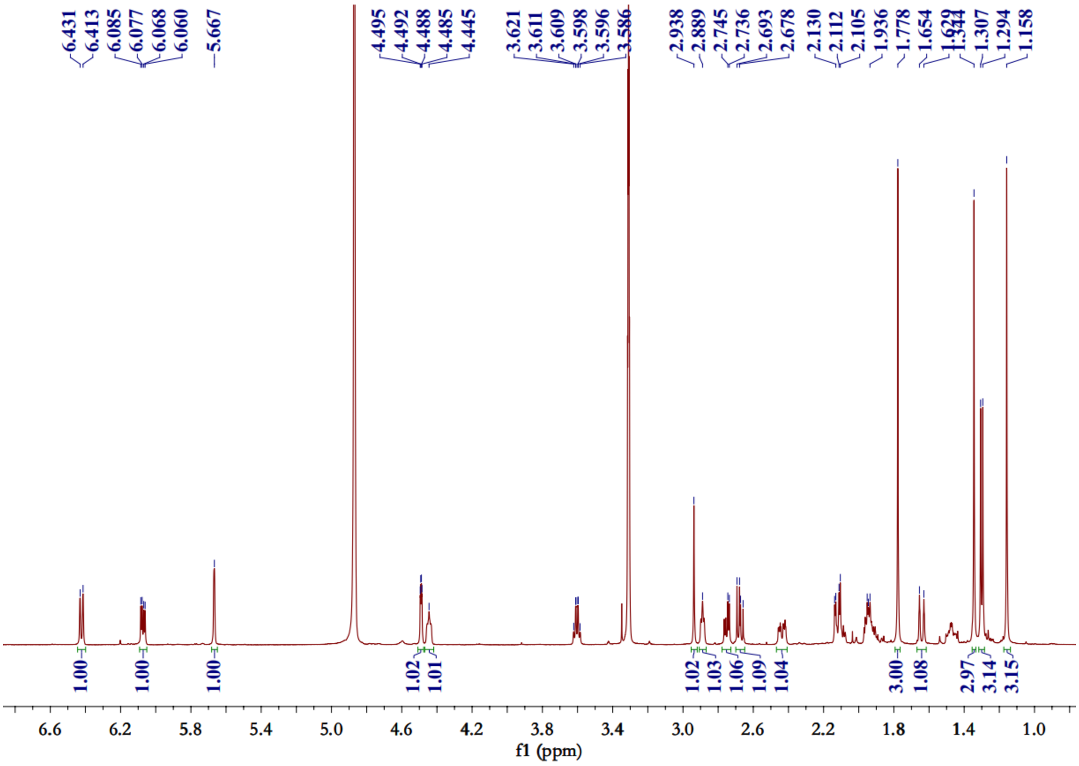

Figure S1. ^{1}H NMR spectrum (600MHz) of compound 1 in CD_3OD.

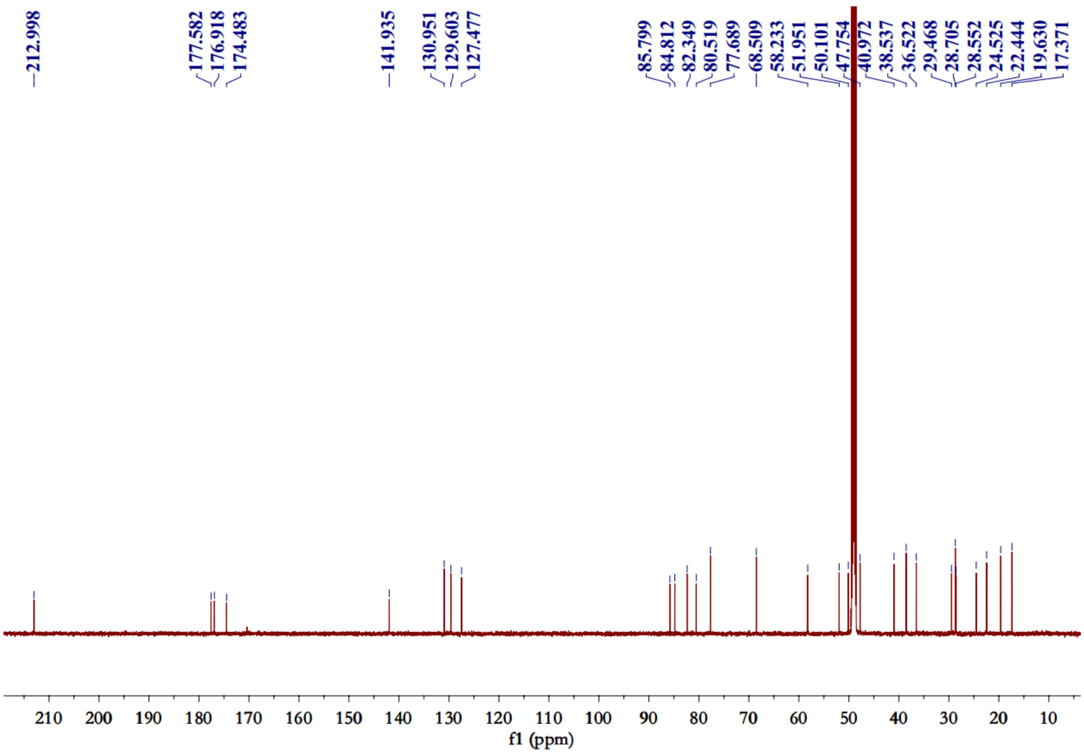

Figure S2. ^{13}C NMR spectrum (150MHz) of compound 1 in CD_3OD.

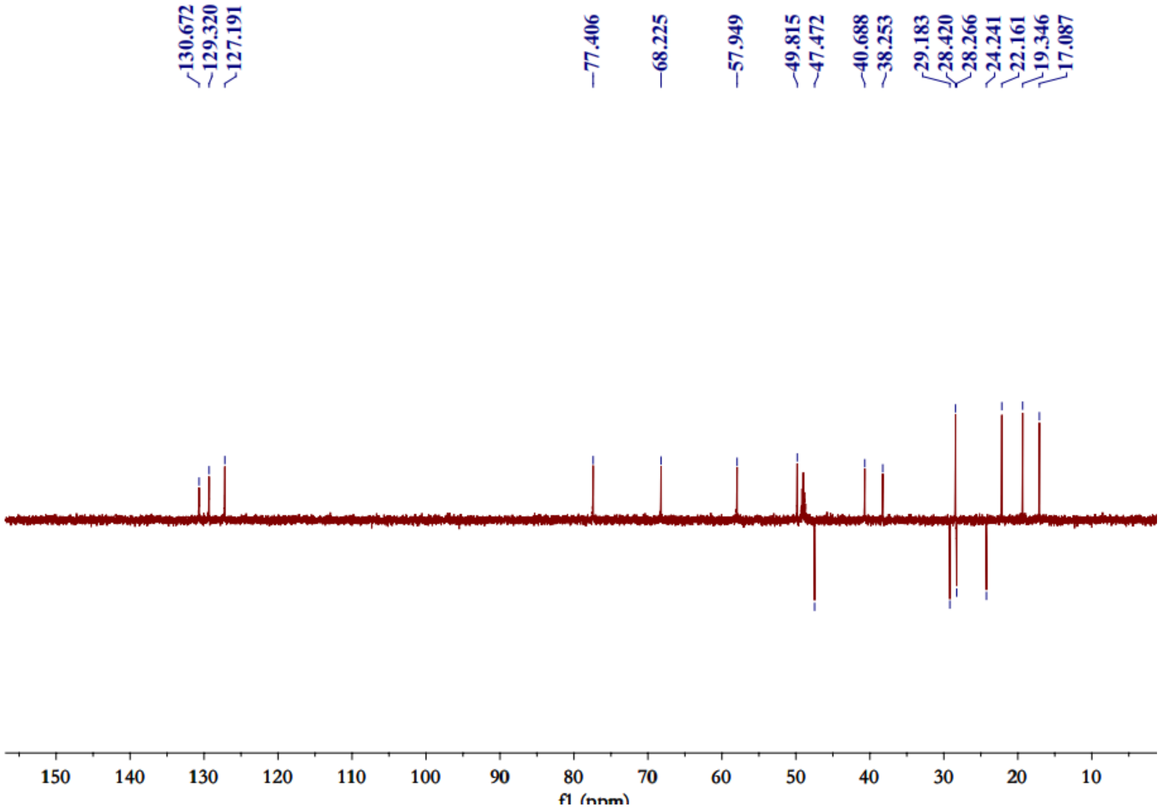

Figure S3. DEPT spectrum (135°) of compound 1 in CD_3OD

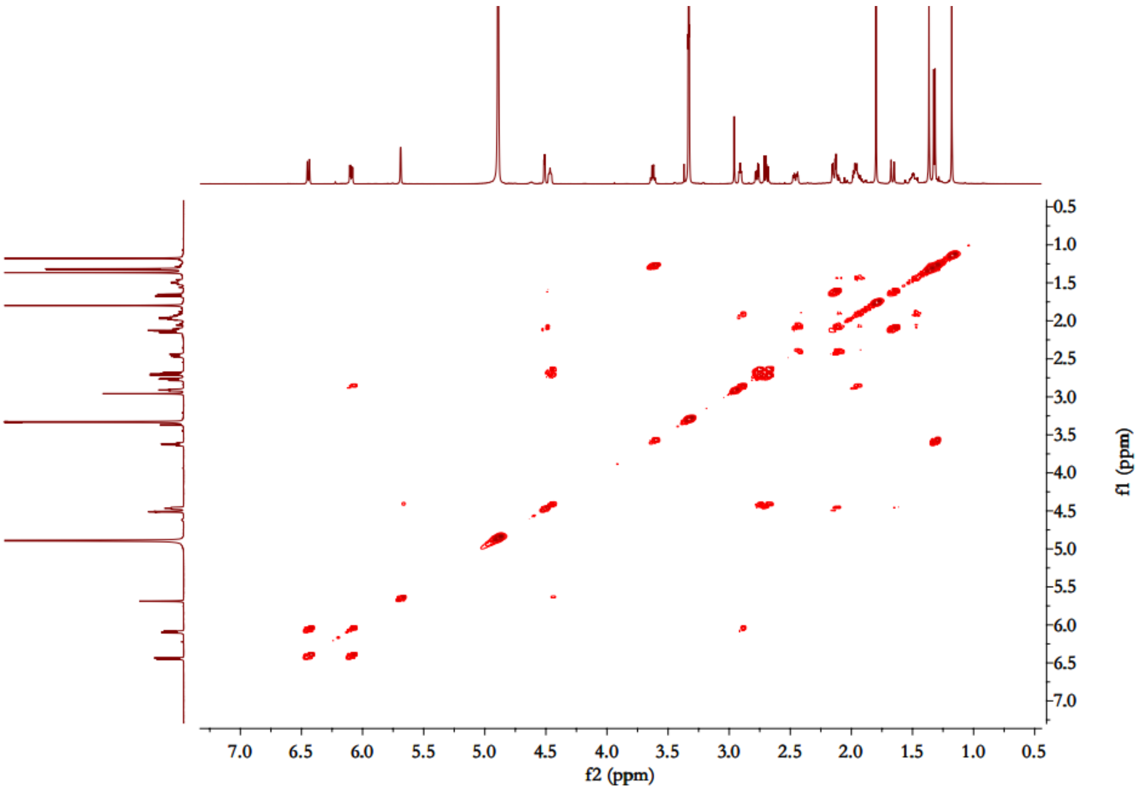

Figure S4. ^1H-^1H COSY spectrum of compound 1 in CD_3OD.

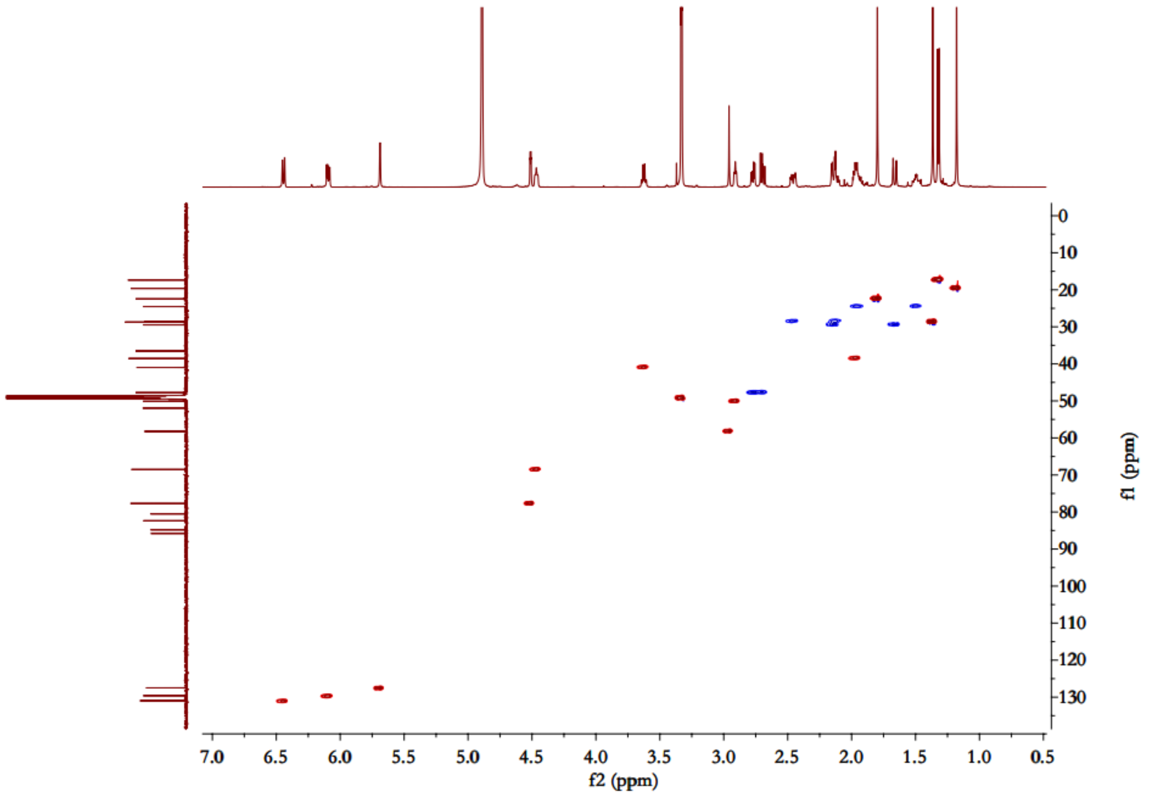

Figure S5 HSQC spectrum of compound 1 in CD_3OD

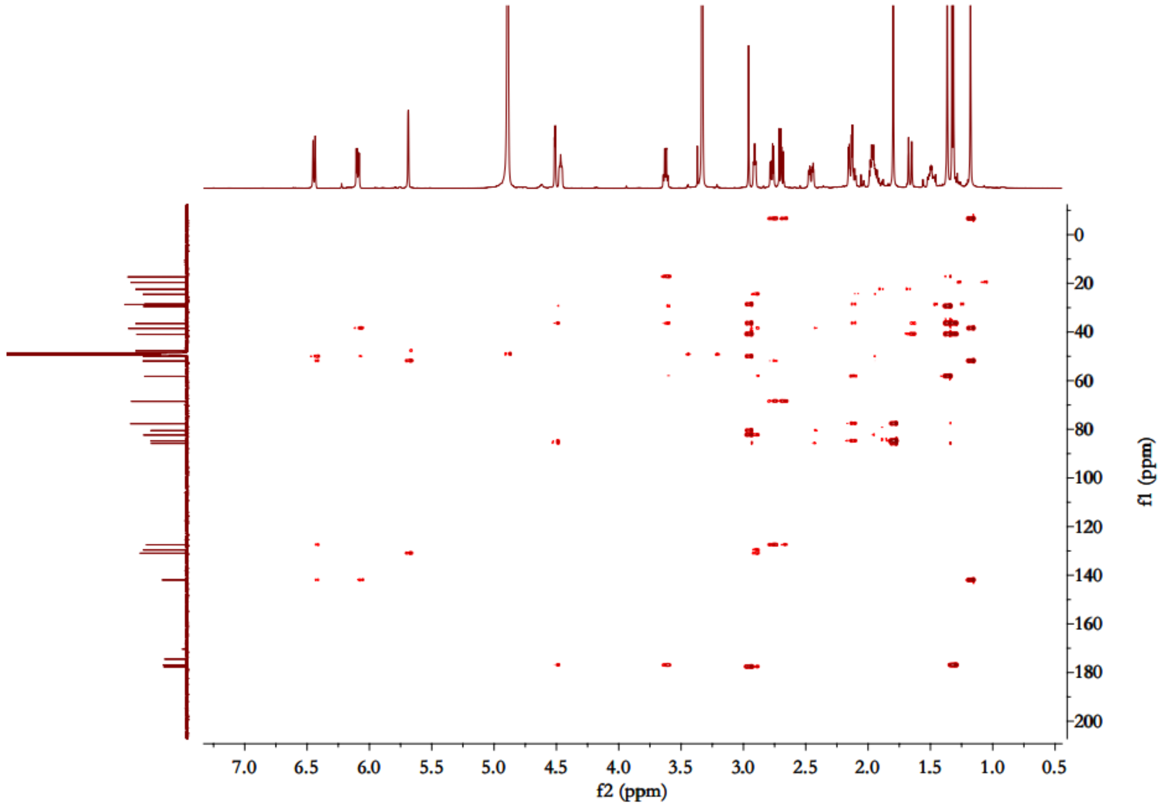

Figure S6. HMBC spectrum of compound 1 in CD_3OD.

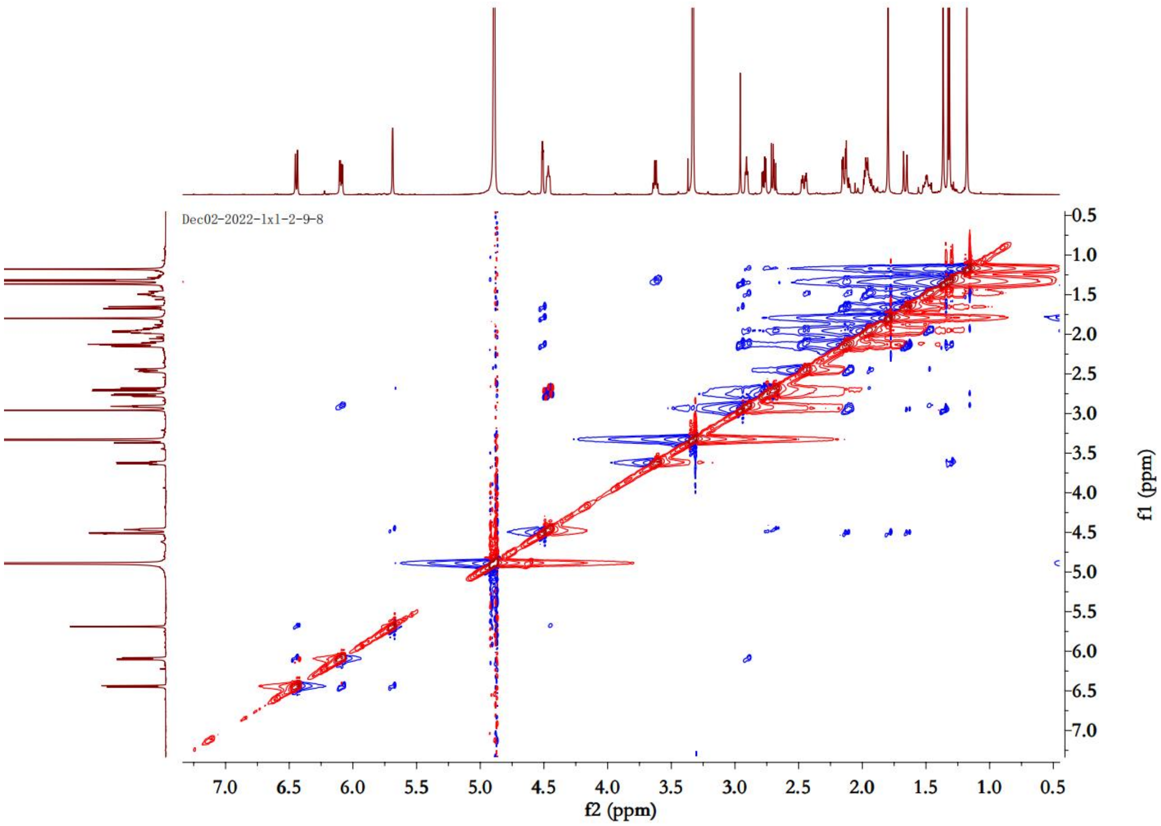

Figure S7. NOSEY spectrum of compound 1 in CD_3OD

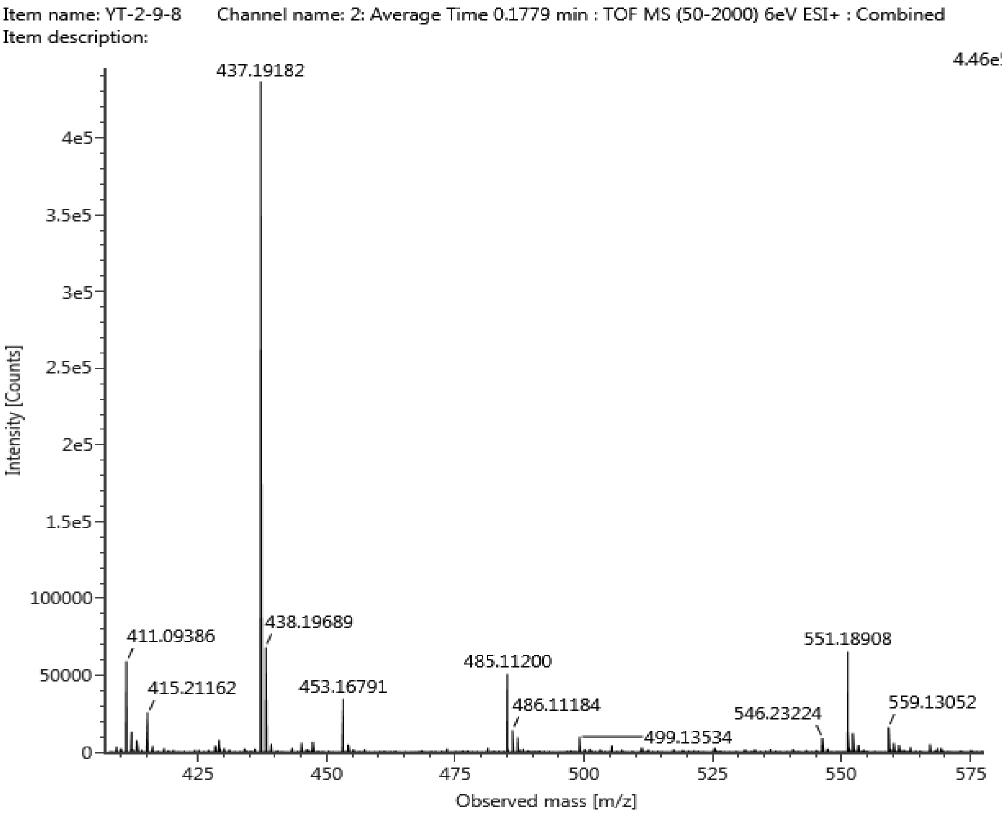

Figure S8. HR-ESI-MS spectrum of compound 1.

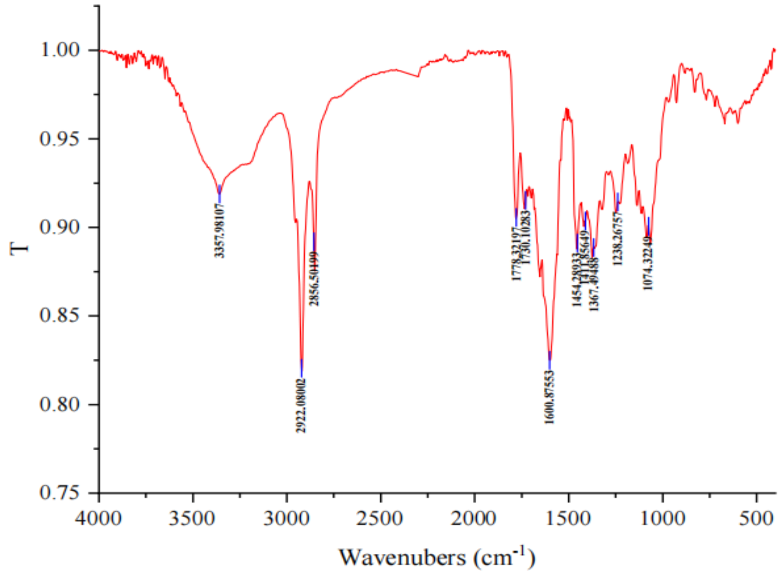

Figure S9. IR spectrum of compound 1.

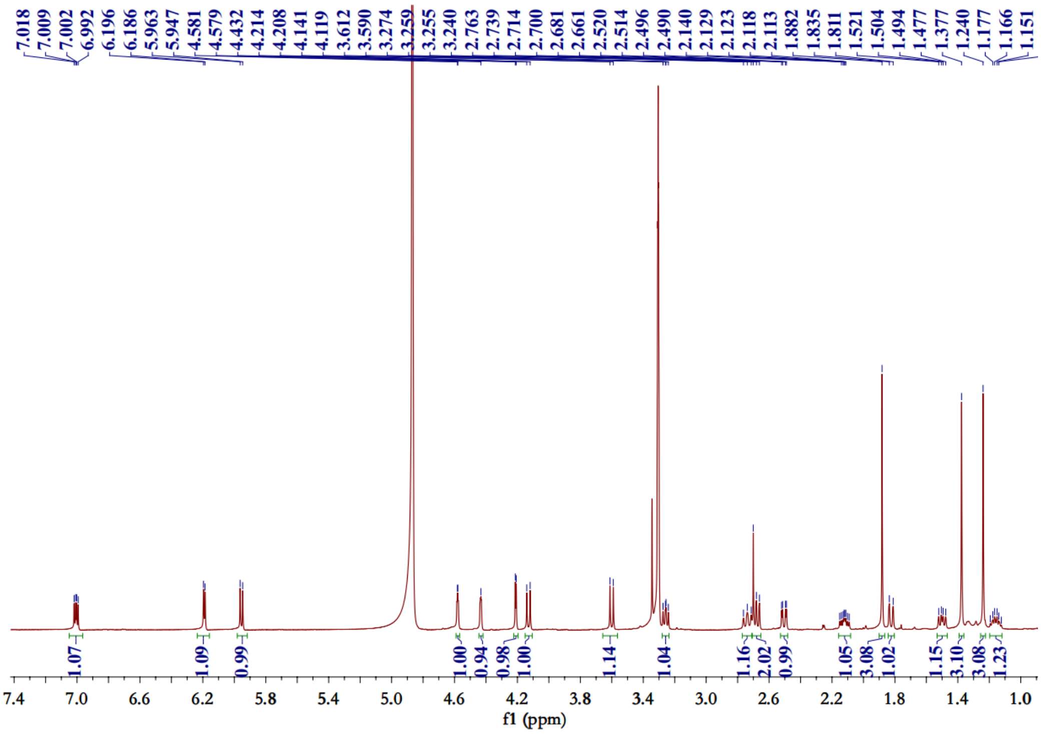

Figure S10. ^{1}H NMR spectrum (600MHz) of compound 2 in CD_3OD.

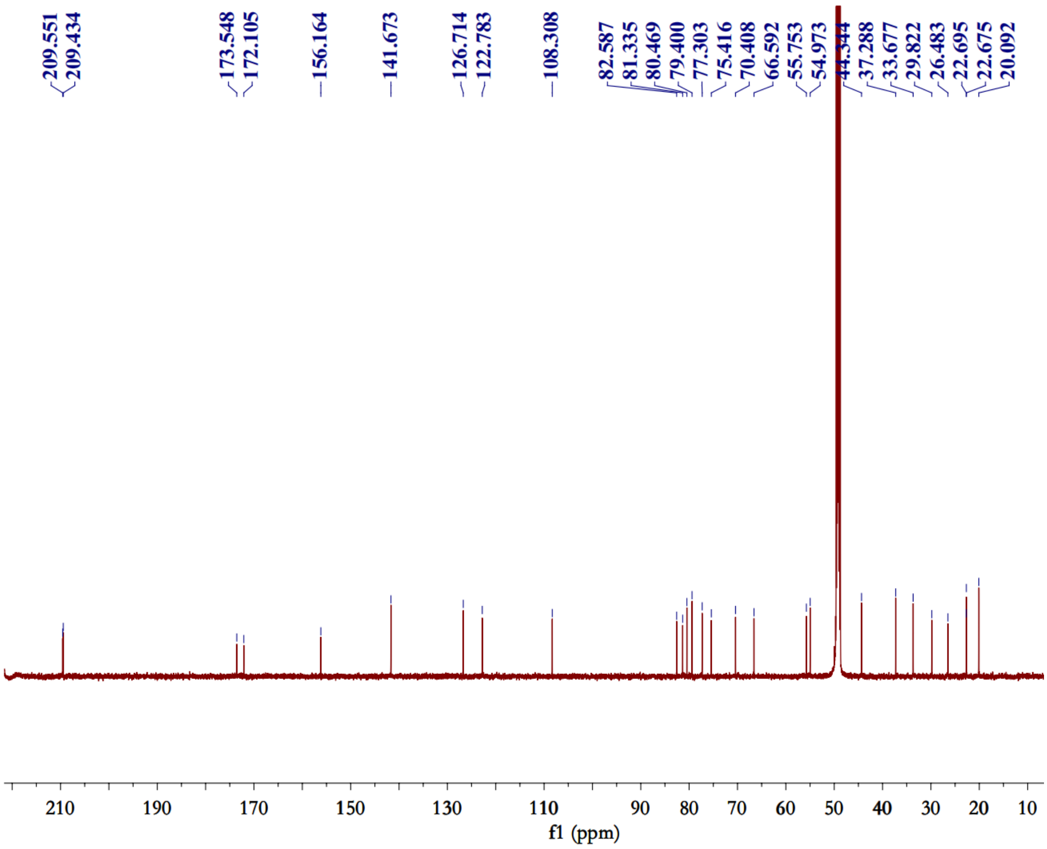

Figure S11. ^{13}C NMR spectrum (150MHz) of compound 2 in CD_3OD.

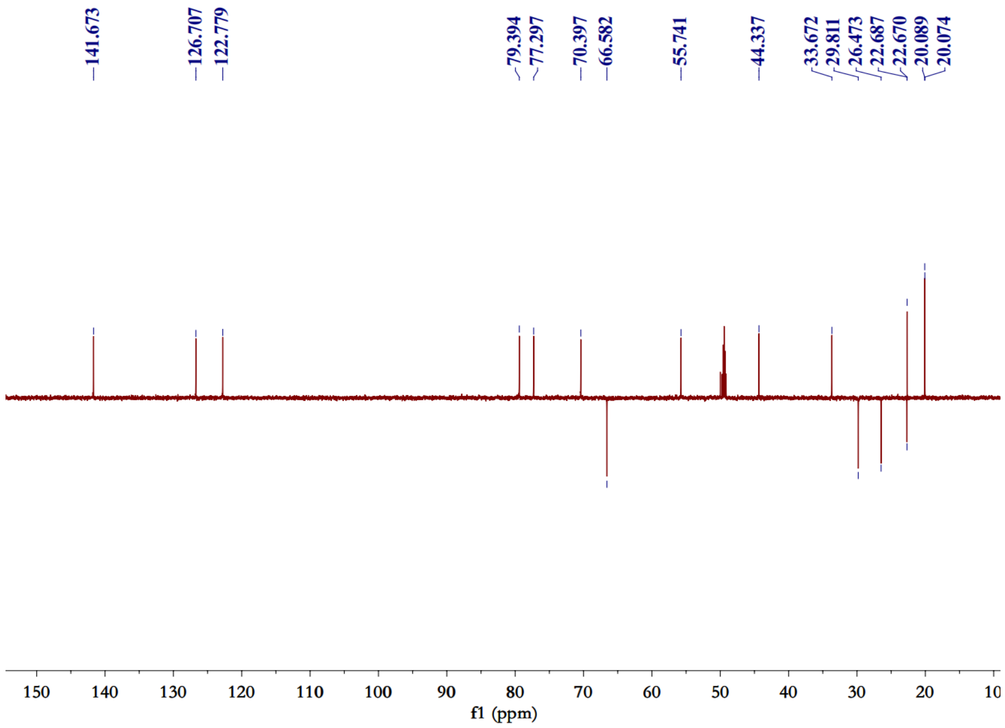

Figure S12. DEPT 135 spectrum of compound 2 in CD_3OD

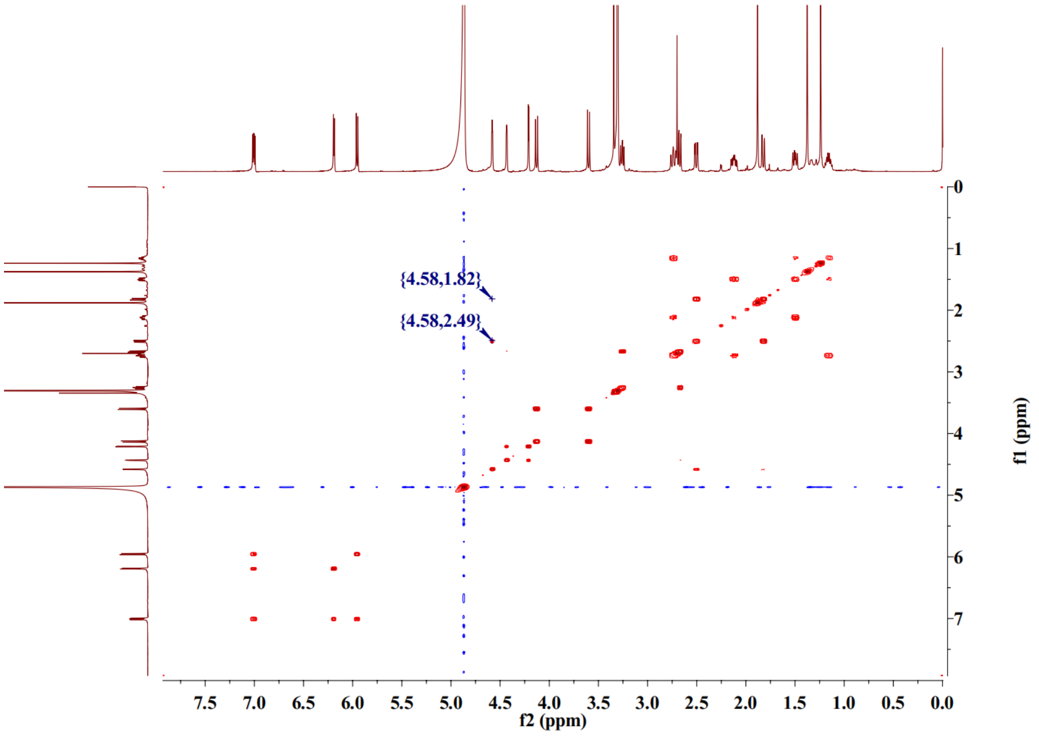

Figure S13. ^{1}H-^{1}H COSY spectrum of compound 2 in CD_3OD.

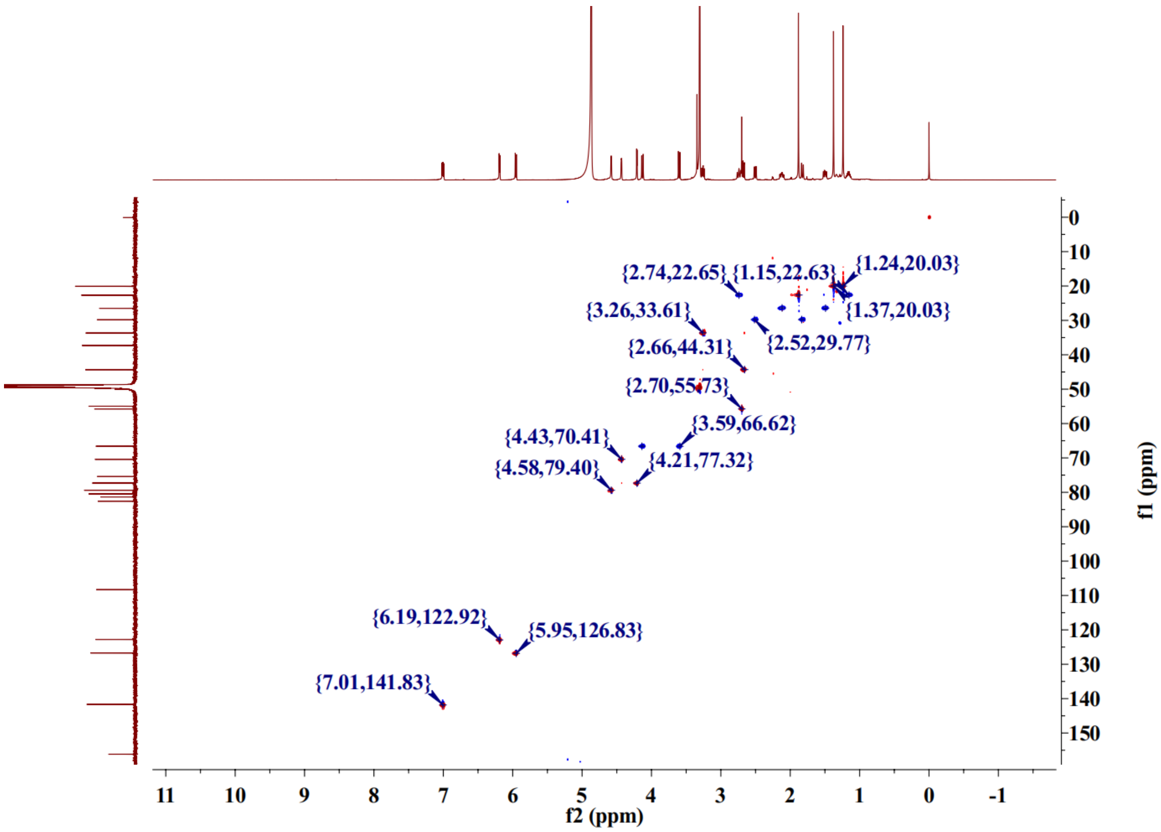

Figure S14. HSQC spectrum of compound 2 in CD_3OD.

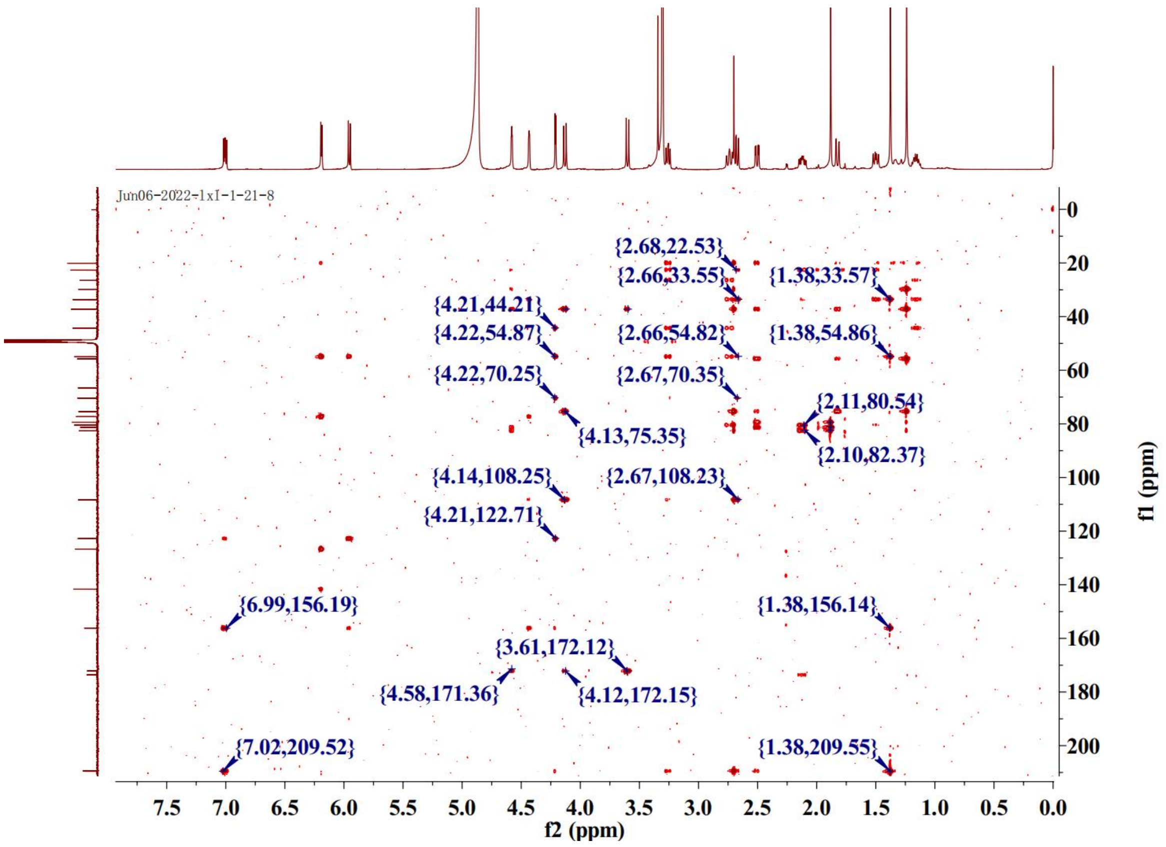

Figure S15. HMBC spectrum of compound 2 in CD_3OD.

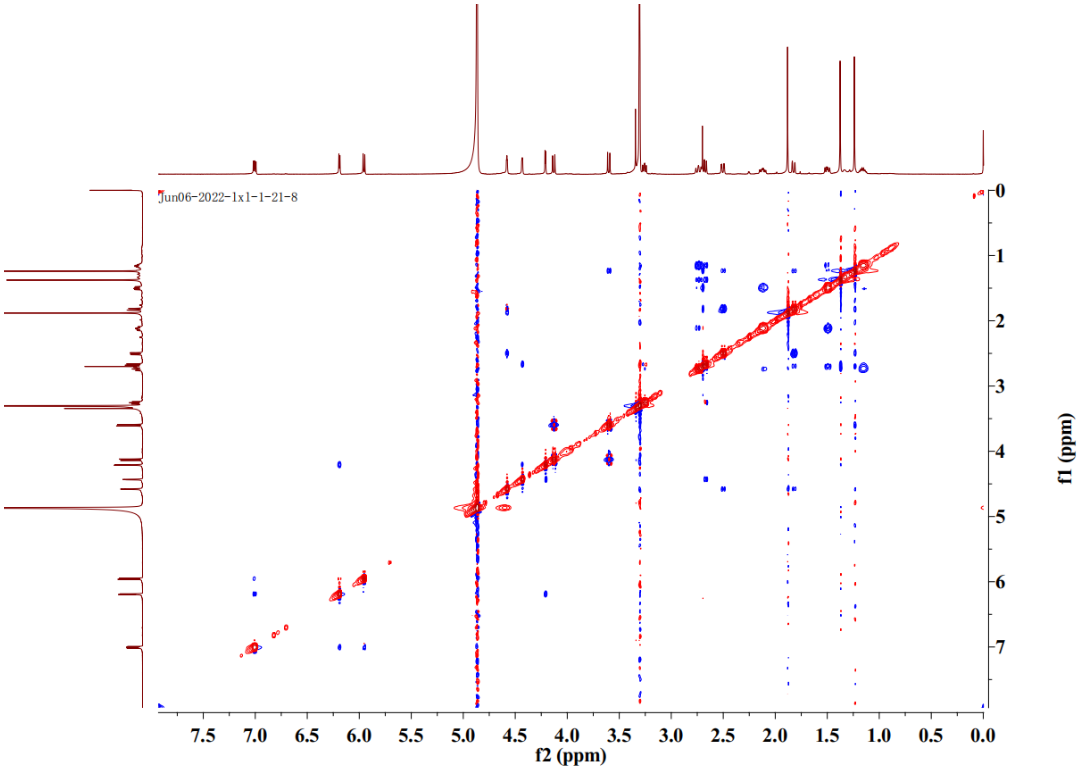

Figure S16. NOSEY spectrum of compound 2 in CD_3OD.

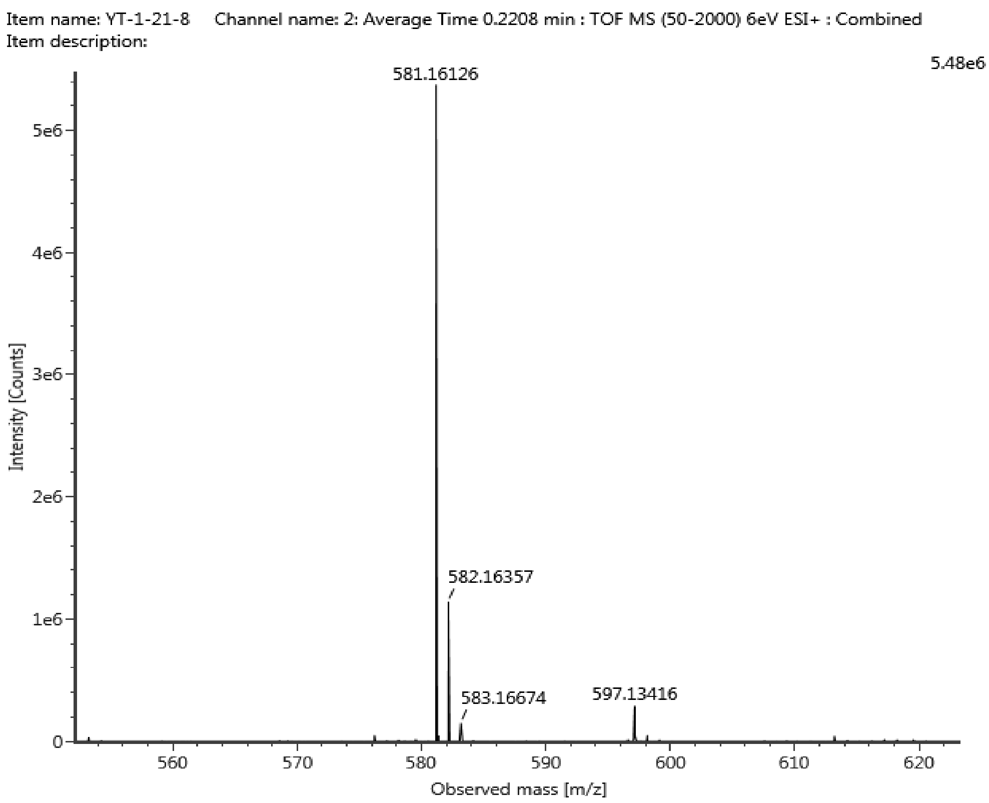

Figure S17 HR-ESI-MS spectrum of compound 2.

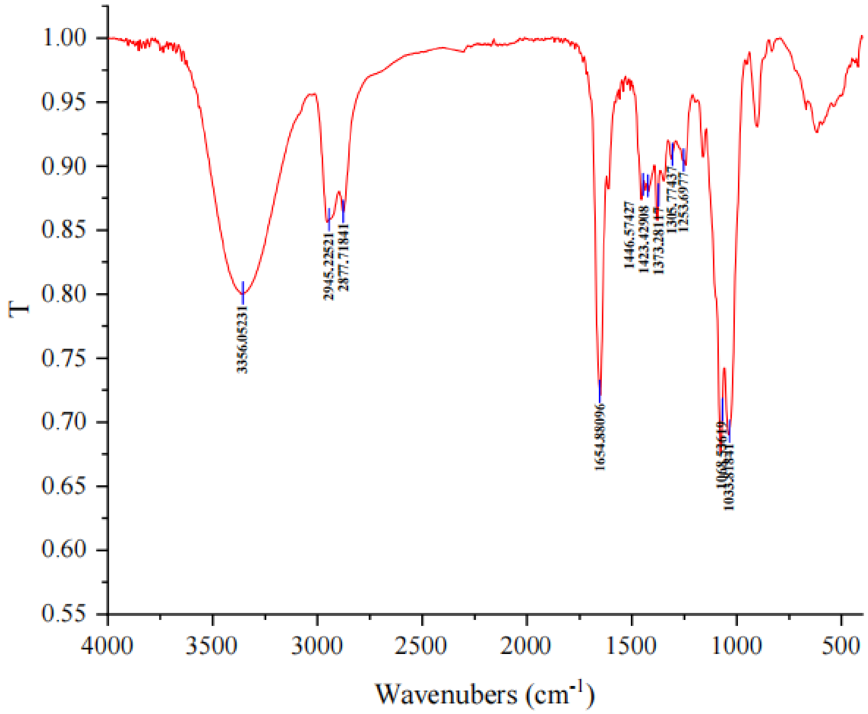

Figure S18. IR spectrum of compound 2

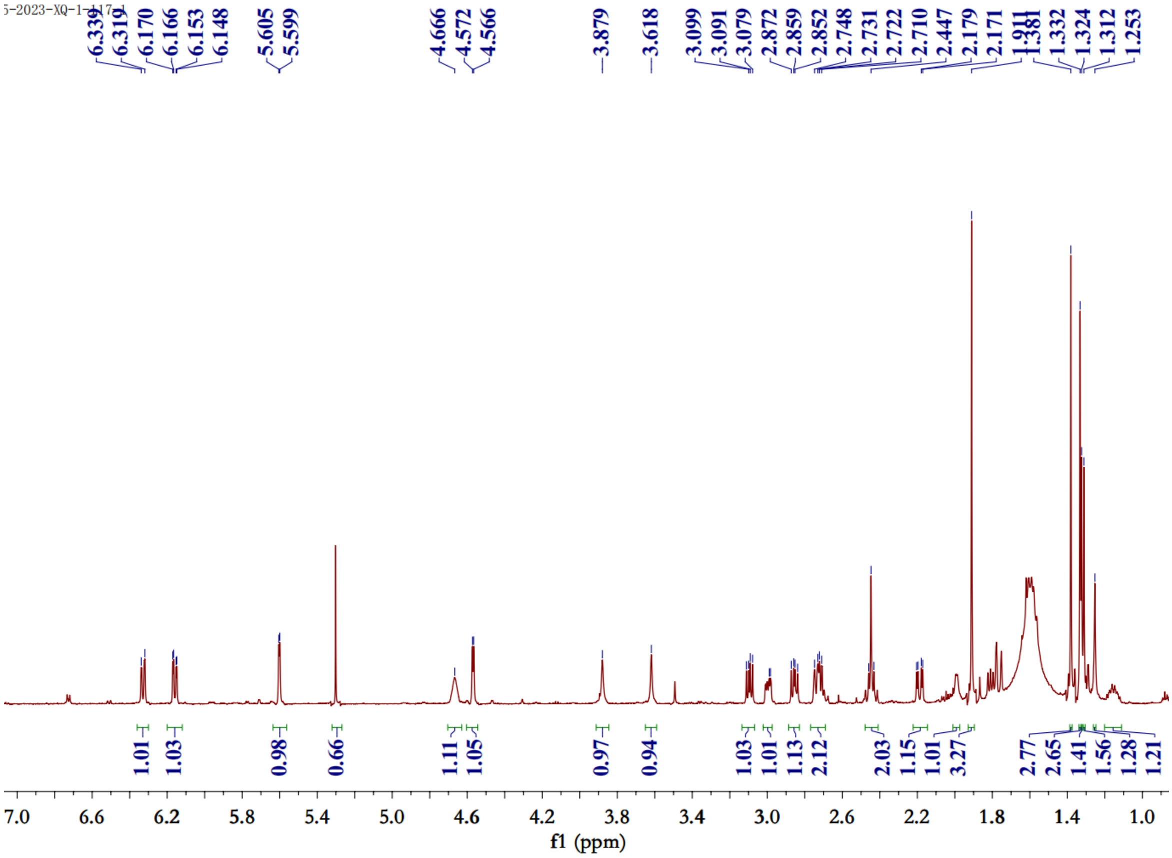

Figure S19. ^{1}H NMR spectrum (600MHz) of compound 3 in $CDCl_3$

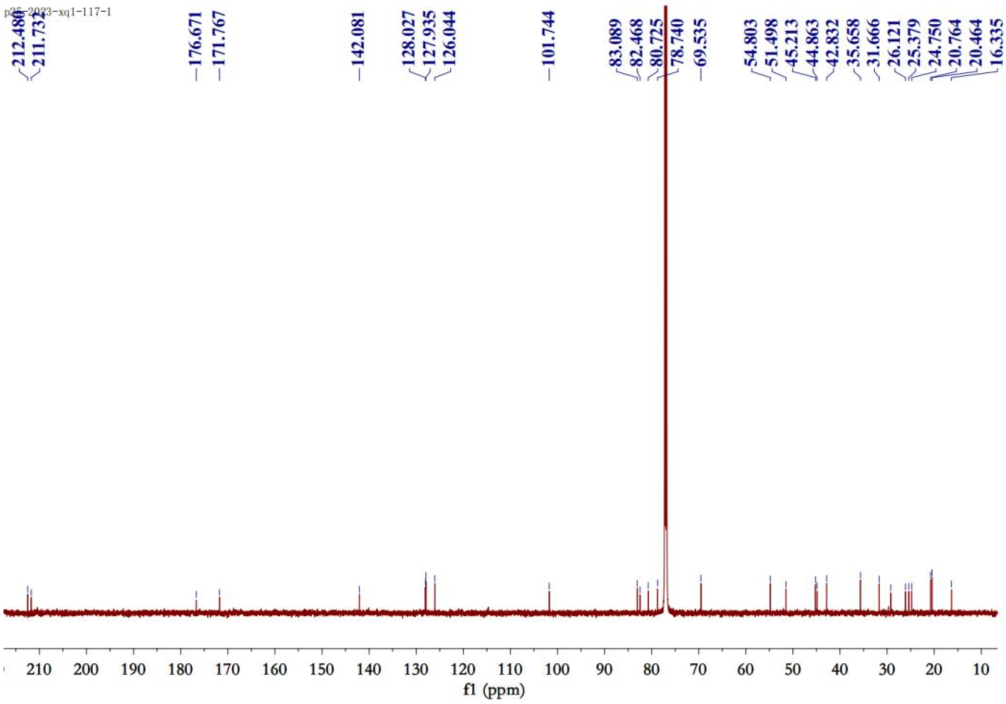

Figure S20. ^{13}C NMR spectrum (150MHz) of compound 3 in $CDCl_3$

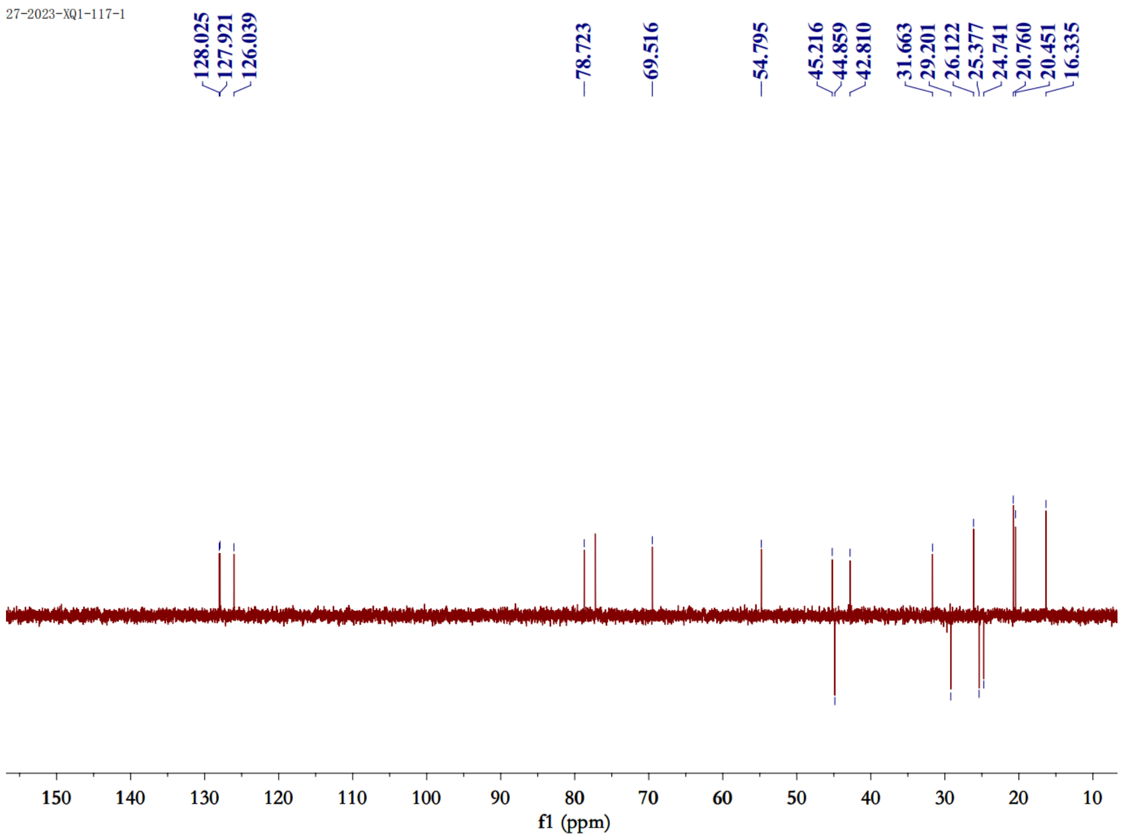

Figure S21. DEPT 135 spectrum of compound 3 in $CDCl_3$

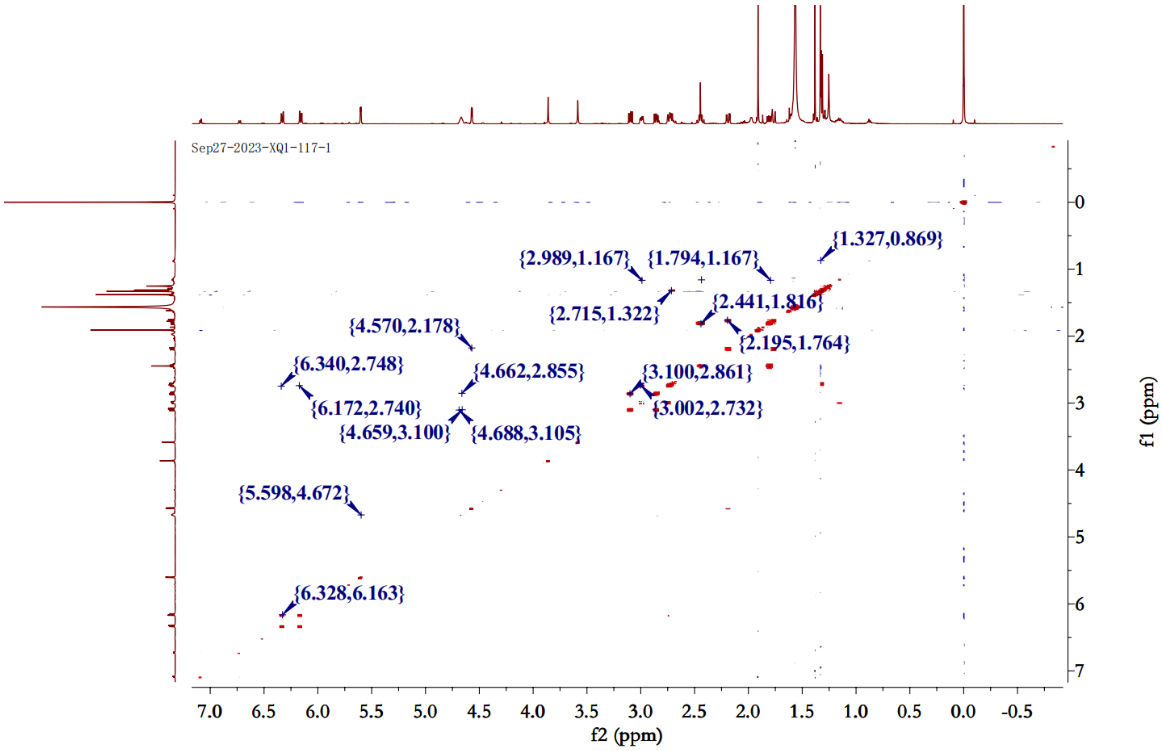

Figure S22. ^{1}H-^{1}H COSY spectrum of compound 3 in $CDCl_3$

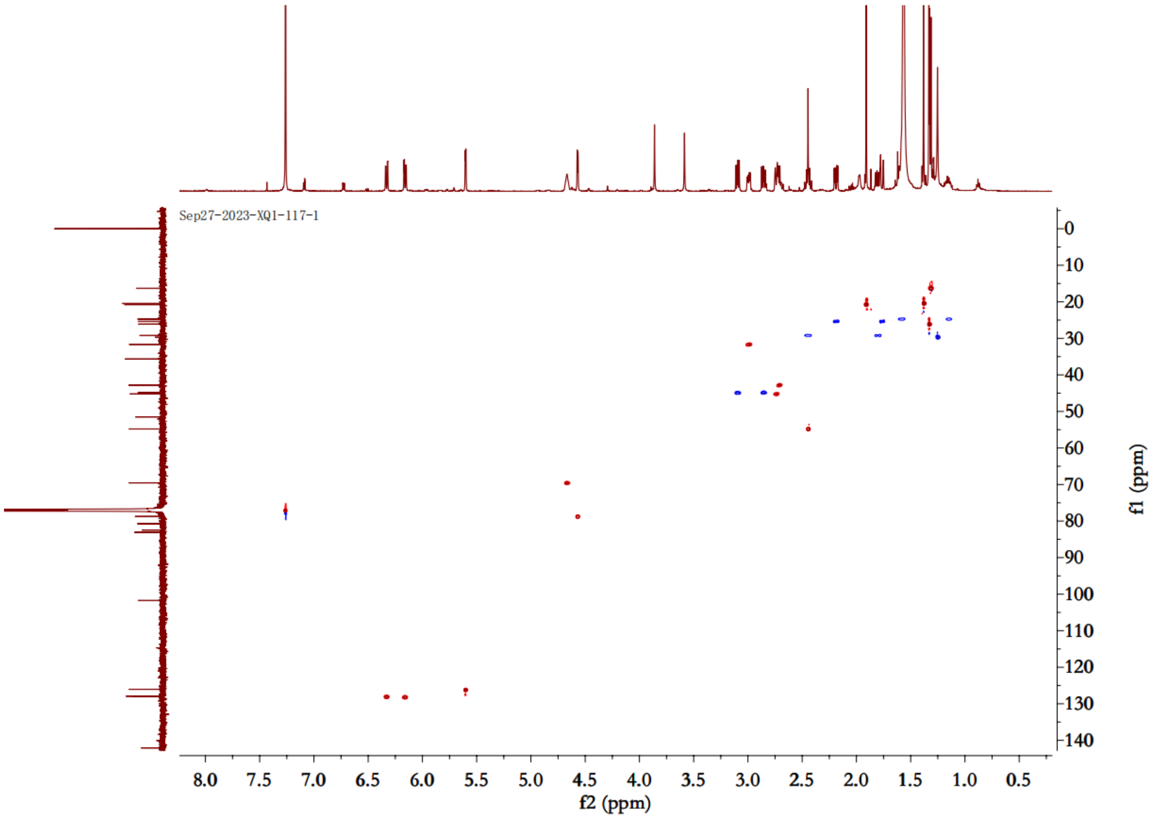

Figure S23. HSQC spectrum of compound 3 in $CDCl_3$

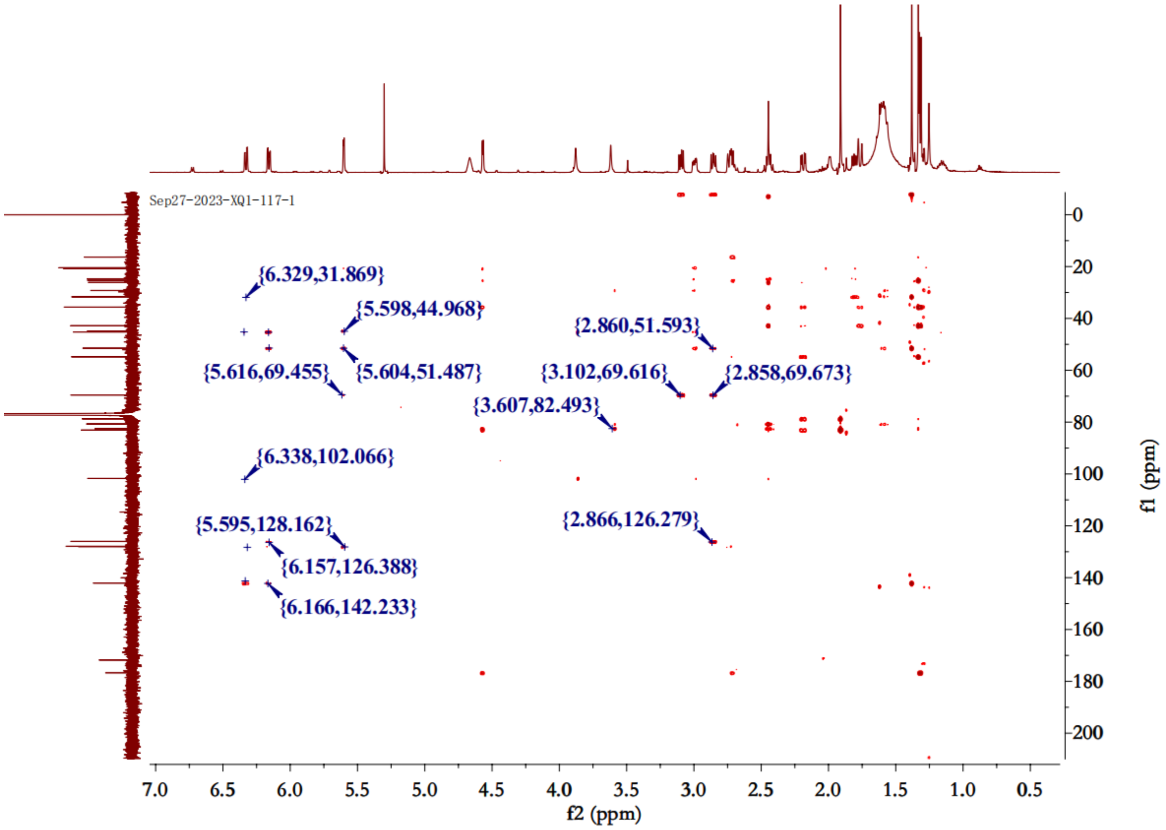

Figure S24. HMBC spectrum of compound 3 in $CDCl_3$

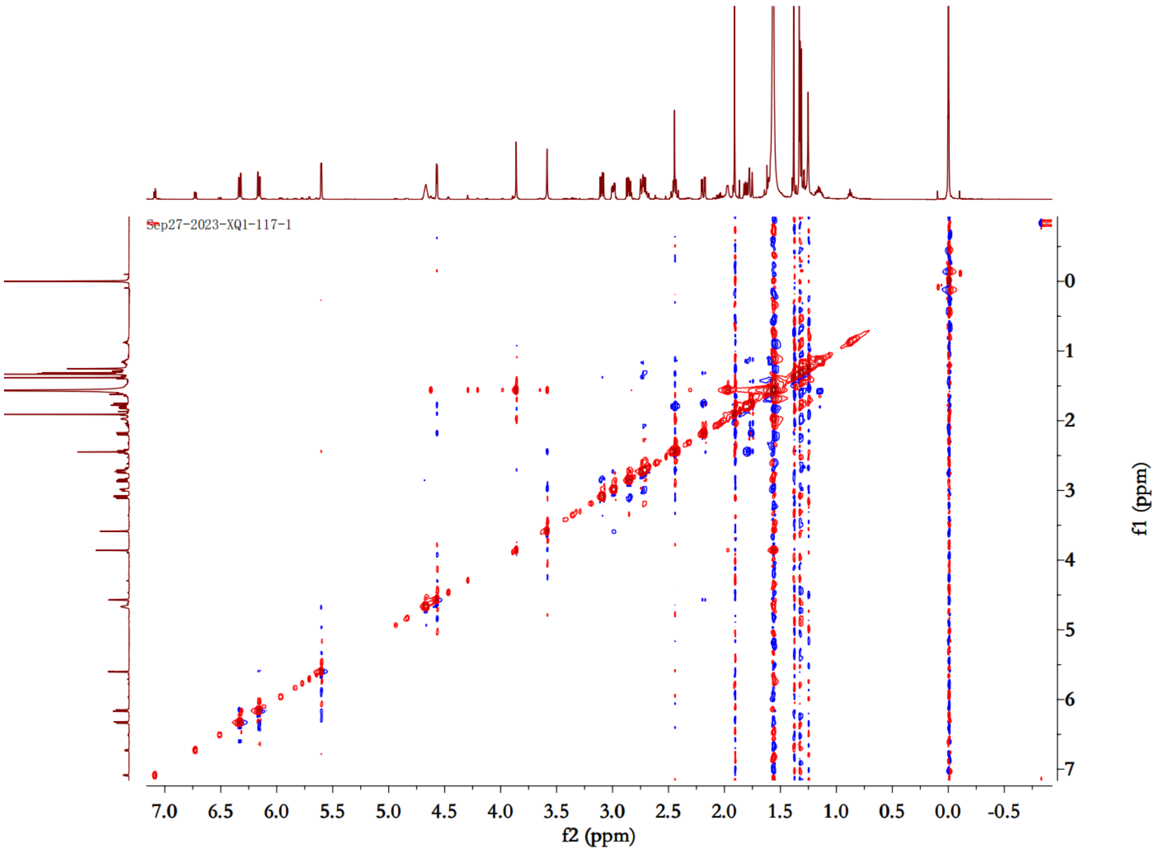

Figure S25. NOSEY spectrum of compound 3 in $CDCl_3$

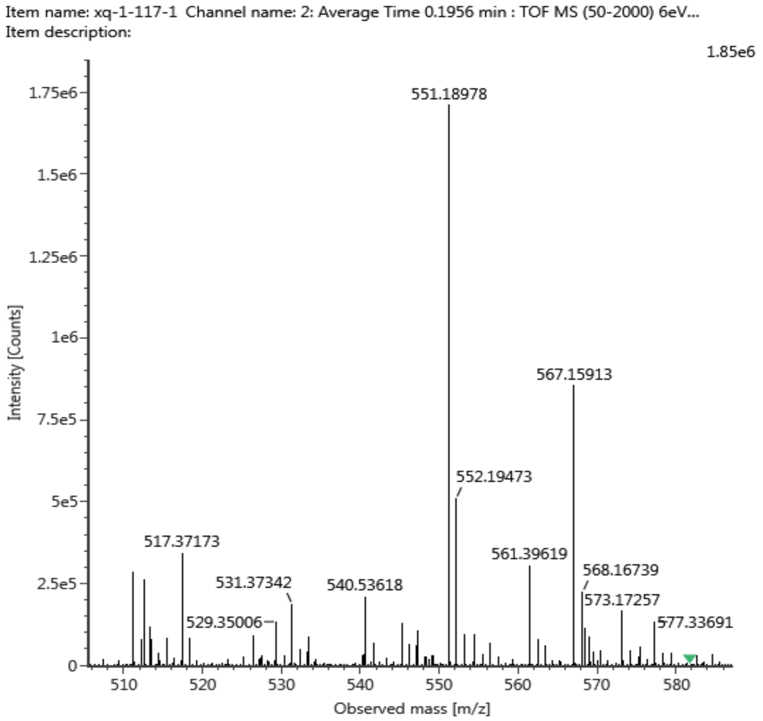

Figure S26. HR-ESI-MS spectrum of compound 3

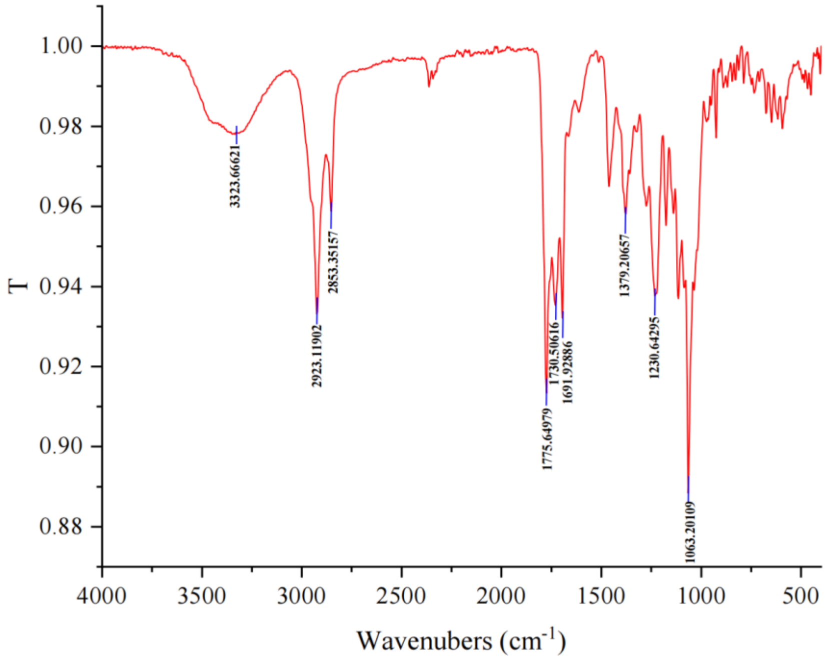

Figure S27. IR spectrum of compound 3

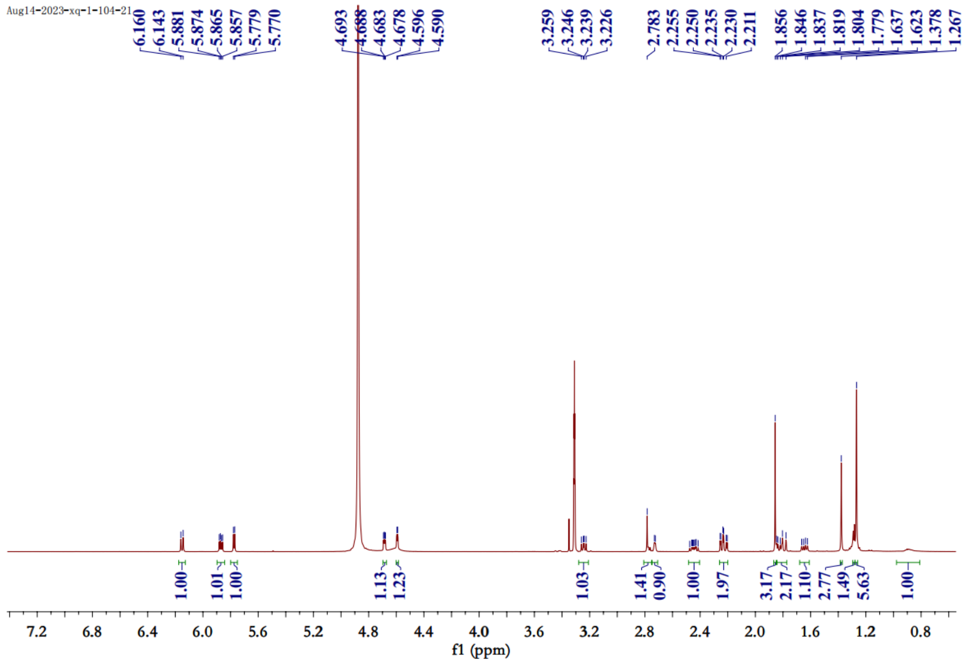

Figure S28. ^{1}H NMR spectrum (600MHz) of compound 4 in CD_3OD

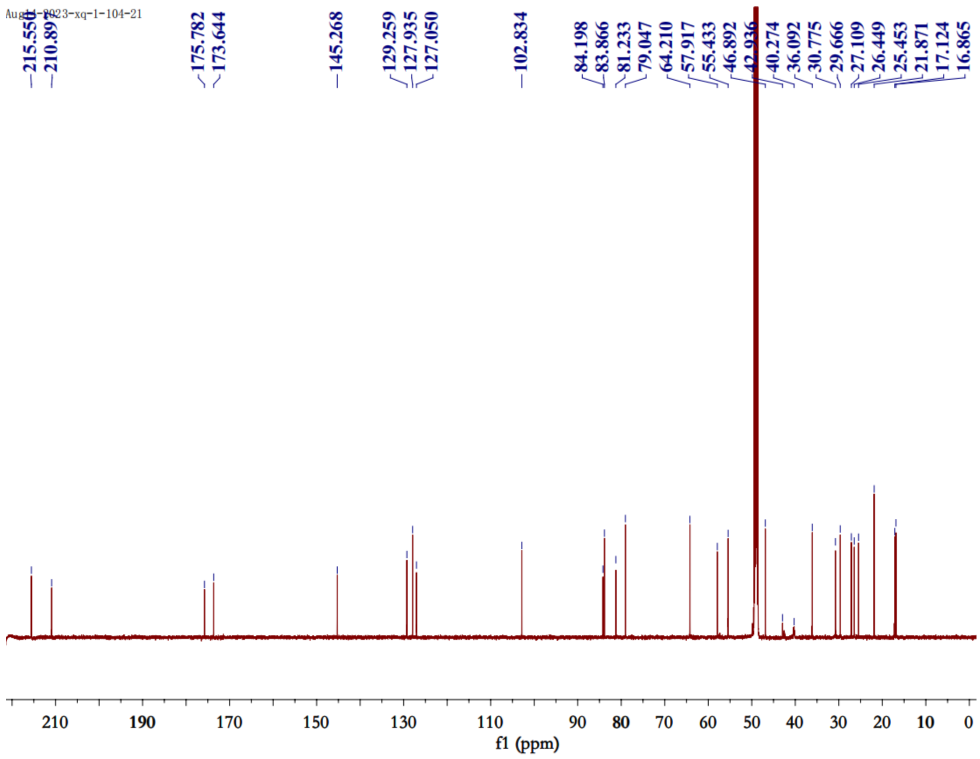

Figure S29. ^{13}C NMR spectrum (150MHz) of compound 4 in CD_3OD

Compound 4:

^{1}H NMR (600 MHz, CD_3OD) δ_H: 6.15(1H, d, *J*= 9.7 Hz, H-4), 5.87(1H, dd, *J*= 9.7, 4.7 Hz, H-3), 5.77(1H, d, *J*= 5.7 Hz, H-6), 4.68(1H, dd, *J*= 5.6, 2.8 Hz, H-7), 4.59(1H, d, *J*= 3.2 Hz, H-22), 3.24(1H, dd, *J*= 12.2, 8.1 Hz, H-9), 2.78(1H, s, H-25), 2.73(1H, d, *J*= 3.8 Hz, H-2), 2.44(1H, m, H-12), 2.23(1H, m, H-8), 1.85(3H, s, H-21), 1.82(1H, m, H-23), 1.64(1H, m, H-11), 1.38(3H, s, H-27), 1.27(6H, s, H-19/H-28).

^{13}C NMR (151 MHz, CD_3OD) δ_C: 215.6(C-15), 210.9(C-1), 175.8(C-18), 173.6(C-26), 145.3(C-5), 129.3(C-4), 127.9(C-6), 127.1(C-3), 102.8(C-14), 84.2(C-13), 83.9(C-17), 81.2(C-20), 79.1(C-22), 64.2(C-7), 57.9(C-10), 55.4(C-16), 46.9(C-8), 42.9(C-2), 40.3(C-25), 36.1(C-24), 30.7(C-12), 29.7(C-9), 27.1(C-23), 26.5(C-21), 25.5(C-11), 21.9(C-19), 17.1(C-27), 16.9(C-28)。

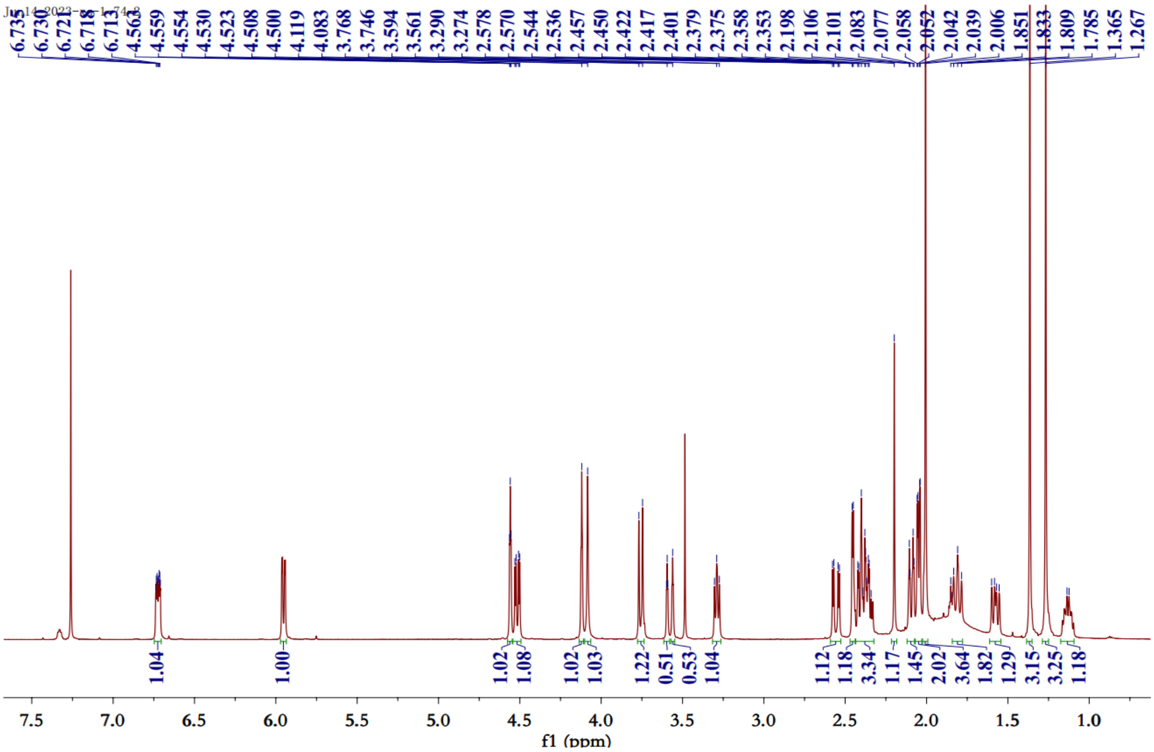

Figure S30. ^{1}H NMR spectrum (600MHz) of compound 5 in $CDCl_3$

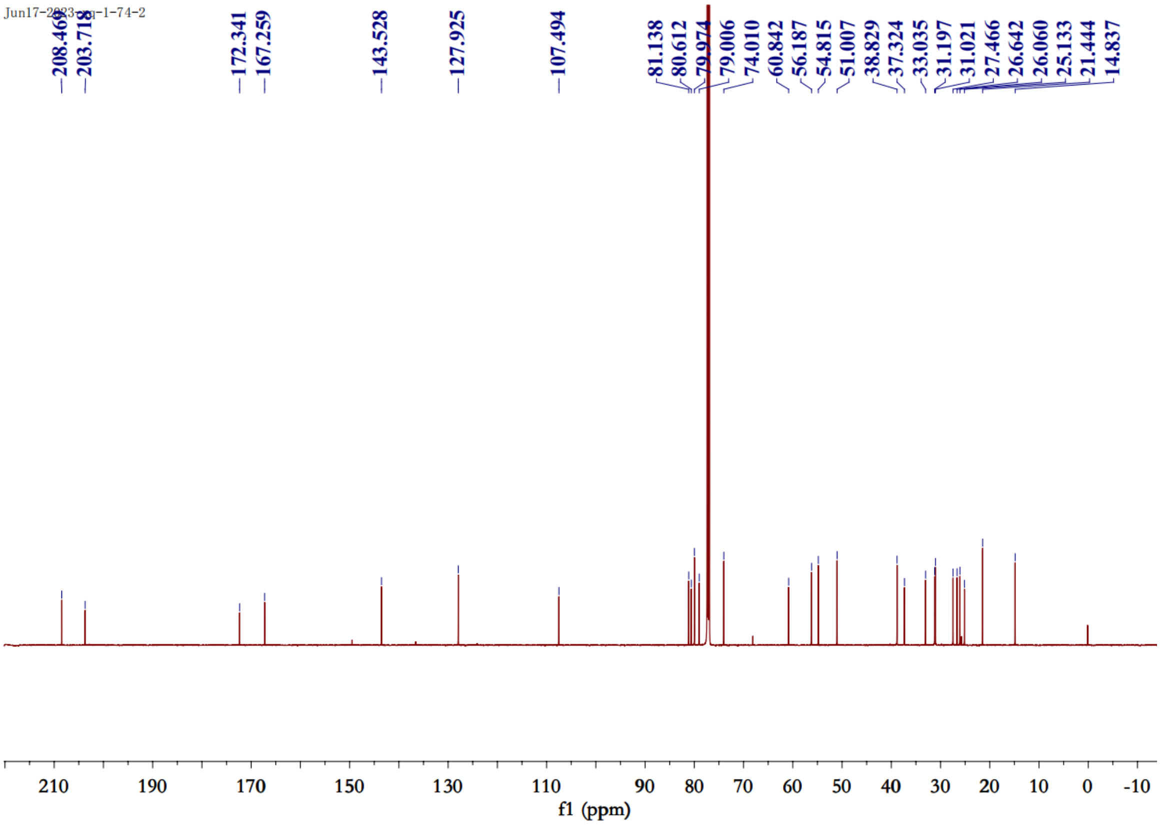

Figure S31. ^{13}C NMR spectrum (150MHz) of compound 5 in $CDCl_3$

Compound 5:

^{1}H NMR (600 MHz, $CDCl_3$) δ_H: 5.95(1H, dd, *J*= 2.57, 10.20 Hz, H-2), 6.72(1H, m, H-3), 2.05(2H, dd, *J*= 2.62, 8.61 Hz, H-4), 3.59(1H, dd, *J*= 10.14, 12.55 Hz, H-6), 1.81(2H, m, H-7), 2.09 (1H, m, H-8), 3.29(1H, m, H-9), 1.13(1H, dd, *J*= 7.55 Hz, H-11a), 1.58(1H, dd, *J*= 10.27, 7.55 Hz, H-11b), 2.40(2H, m, H-12), 2.20(1H, s, H-16), 1.27 (3H, s, H-19), 2.38(3H, s, H-21), 4.56(1H, t, *J* = 2.65 Hz, H-22), 2.35(1 H, dd, *J*= 4.26, 11.93 Hz, H-23a), 2.45 (1H, d, *J*= 4.26 Hz, H-23b), 4.52(1H, dd, *J*= 4.60, 13.50 Hz, H-27a), 3.76(1H, d, *J*= 13.50 Hz, H-27b), 1.37(3H, s, H-28).

^{13}C NMR (151 MHz, $CDCl_3$) δ_C: 203.7(C-1), 127.9(C-2), 143.5(C-3), 37.3(C-4), 79.3(C-5), 74.0(C-6), 27.5(C-7), 38.8(C-8), 31.2(C-9), 56.2(C-10), 26.1(C-11), 80.0(C-13), 26.6(C-12), 107.5(C-14), 208.5(C-15), 54.8(C-16), 81.1(C-17), 172.3(C-18), 14.8(C-19), 80.6(C-20), 21.4(C-21), 77.0(C-22), 33.0(C-23), 31.0(C-24), 51.0(C-25), 167.3(C-26), 60.8(C-27), 25.1(C-28)。

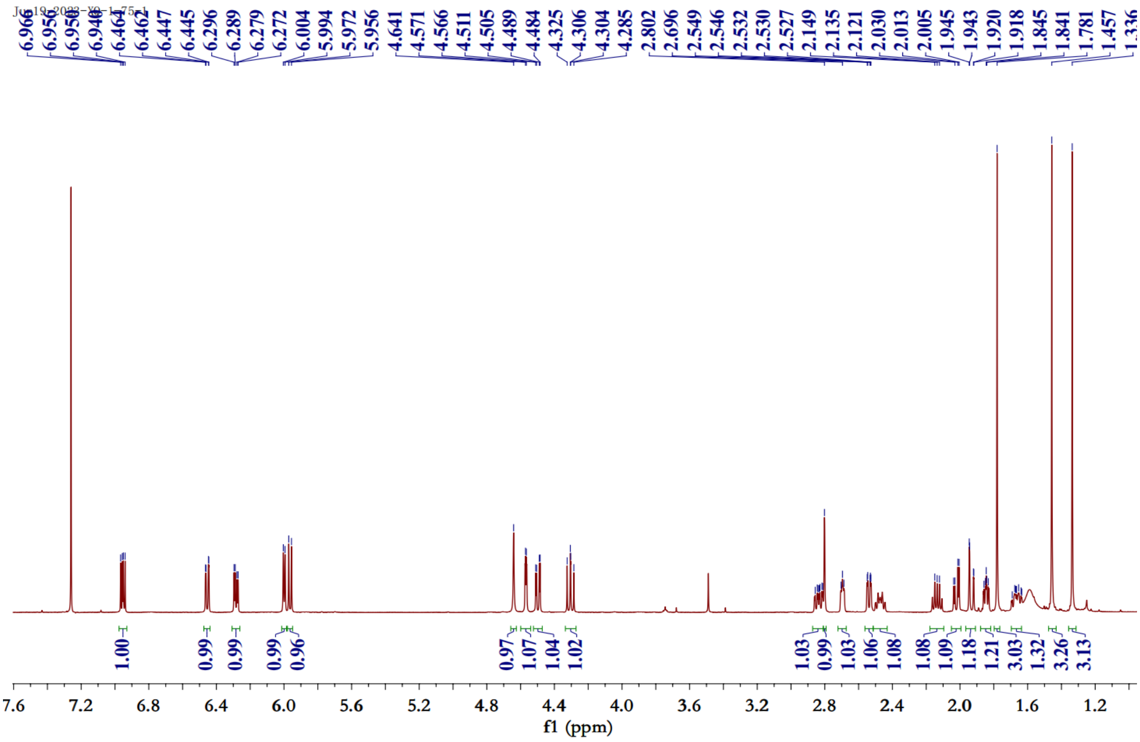

Figure S32. ^{1}H NMR spectrum (600MHz) of compound 6 in $CDCl_3$

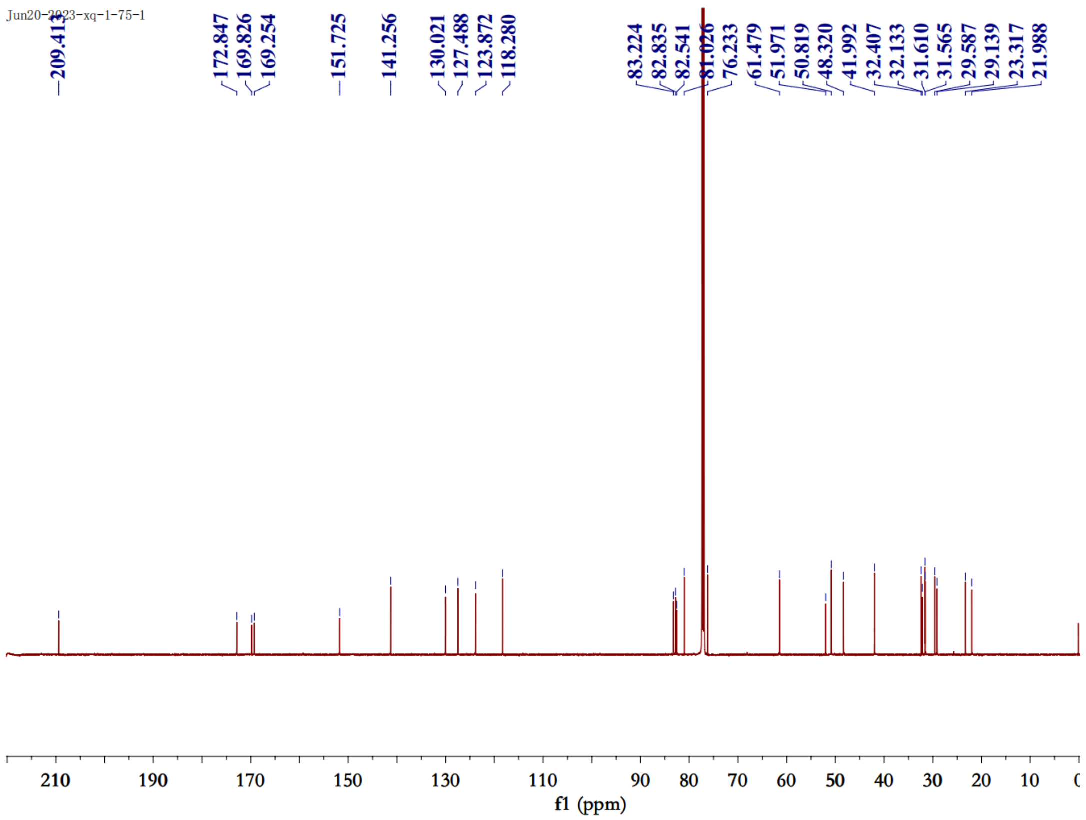

Figure S33. ^{13}C NMR spectrum (150MHz) of compound 6 in $CDCl_3$

Compound 6:

^{1}H NMR (600 MHz, $CDCl_3$) δ_H: 6.00(1H, d, *J*= 6.10 Hz, H-2), 6.95(1H, d, *J*= 6.10, 9.70 Hz, H-3), 5.96(1H, d, *J*=9.70 Hz, H-4), 6.45(1H, dd, *J*= 1.19, 10.20 Hz, H-6), 6.28(1H, dd, *J*= 4.22, 10.20Hz, H-7), 2.83(1H, m, H-8), 1.85(1H, m, H-9), 2.70(1H, m, H-11a), 2.47(1H, m, H-11b), 2.14(1H, m, H-12a), 1.85(1H, m, H-12b), 2.80(1H, s, H-16), 1.34(3H, s, H-19), 1.78(3H, s, H-21), 4.57(1H, d, *J*= 2.99 Hz, H-22), 1.93(1H, dd, *J*= 1.30, 14.94 Hz, H-23a), 2.02(1H, dd, *J*= 4.46, 14.94 Hz, H-23b), 2.54(1H, dd, *J*= 1.75, 11.25 Hz, H-25), 4.50(1H, dd, *J*= 3.04, 12.55 Hz, H-26a), 4.30(1H, dd, *J*= 11.34, 12.55 Hz, H-26b), 1.46(3H, s, H-28).

^{13}C NMR (151 MHz, $CDCl_3$) δ_C: 209.4(C-1), 119.3(C-2), 141.3(C-3), 123.9(C-4), 151.7(C-5), 127.5(C-6), 130.0(C-7), 50.8(C-8), 32.4(C-9), 52.0(C-10), 31.6(C-11), 29.4(C-12), 81.30(C-13), 82.5(C-14), 169.8(C-15), 48.3(C-16), 82.5(C-17), 172.8(C-18), 23.3(C-19), 83.2(C-20), 22.0(C-21), 76.2(C-22), 31.6(C-23), 29.6(C-24), 42.0(C-25), 61.5(C-26), 169.2(C-27), 31.6(C-28).
